# Pore Forming Protein Induced Biomembrane Reorganization and Dynamics: A Focused Review

**DOI:** 10.3389/fmolb.2021.737561

**Published:** 2021-09-09

**Authors:** Ilanila Ilangumaran Ponmalar, Nirod K. Sarangi, Jaydeep K. Basu, K. Ganapathy Ayappa

**Affiliations:** ^1^ Center for BioSystems Science and Engineering, Indian Institute of Science, Bangalore, India; ^2^ School of Chemical Science, Dublin City University, Dublin, Ireland; ^3^ Department of Physics, Indian Institute of Science, Bangalore, India; ^4^ Department of Chemical Engineering, Indian Institute of Science, Bengaluru, India

**Keywords:** pore-forming toxin, listeriolysin O, cytolysin A, lipid dynamics, fluorescence correlation spectroscopy, STED nanoscopy, molecular dynamics simulations

## Abstract

Pore forming proteins are a broad class of pathogenic proteins secreted by organisms as virulence factors due to their ability to form pores on the target cell membrane. Bacterial pore forming toxins (PFTs) belong to a subclass of pore forming proteins widely implicated in bacterial infections. Although the action of PFTs on target cells have been widely investigated, the underlying membrane response of lipids during membrane binding and pore formation has received less attention. With the advent of superresolution microscopy as well as the ability to carry out molecular dynamics (MD) simulations of the large protein membrane assemblies, novel microscopic insights on the pore forming mechanism have emerged over the last decade. In this review, we focus primarily on results collated in our laboratory which probe dynamic lipid reorganization induced in the plasma membrane during various stages of pore formation by two archetypal bacterial PFTs, cytolysin A (ClyA), an *α*-toxin and listeriolysin O (LLO), a *β*-toxin. The extent of lipid perturbation is dependent on both the secondary structure of the membrane inserted motifs of pore complex as well as the topological variations of the pore complex. Using confocal and superresolution stimulated emission depletion (STED) fluorescence correlation spectroscopy (FCS) and MD simulations, lipid diffusion, cholesterol reorganization and deviations from Brownian diffusion are correlated with the oligomeric state of the membrane bound protein as well as the underlying membrane composition. Deviations from free diffusion are typically observed at length scales below ∼130 nm to reveal the presence of local dynamical heterogeneities that emerge at the nanoscale—driven in part by preferential protein binding to cholesterol and domains present in the lipid membrane. Interrogating the lipid dynamics at the nanoscale allows us further differentiate between binding and pore formation of *β*- and *α*-PFTs to specific domains in the membrane. The molecular insights gained from the intricate coupling that occurs between proteins and membrane lipids and receptors during pore formation are expected to improve our understanding of the virulent action of PFTs.

## 1 Introduction

A large class of bacterial pathogens have evolved to infect target cells by releasing pore forming membrane disruptive proteins whose sole purpose is to compromise metabolic and signaling pathways leading to cell lysis (Morton et al., 2019). Pore-forming toxins (PFTs), a class of membrane excision causing proteins, are generally secreted by pathogenic bacteria as a virulence factor for attacking the host or as a defense mechanism to guard against the host immune response (Dal Peraro and van der Goot, 2016; Morton et al., 2019). Since the plasma membrane is the first point of interrogation during the lytic pathway of PFTs, understanding the dynamic response mediated by these virulent proteins plays an important part in the development of our understanding of virulent bacterial infections (Rojko and Anderluh, 2015). Unlike integral membrane proteins ubiquitously present in the mammalian cell membranes, PFTs undergo dynamic oligomerization and membrane insertion events to create transmembrane oligomeric assemblies that disrupt the integrity of the membrane in a myriad of ways. Discerning features are the active membrane remodeling and lipid ejection (Leung et al., 2014; Vögele et al., 2019; Desikan et al., 2020; Ilanila et al., 2021) events which occur during pore formation. Although the lytic pathways, structure of the pore assemblies and biochemical basis for pore formation has been extensively investigated, the underlying dynamic response of the plasma membrane has only recently been the subject of greater scrutiny.

**FIGURE 1:**
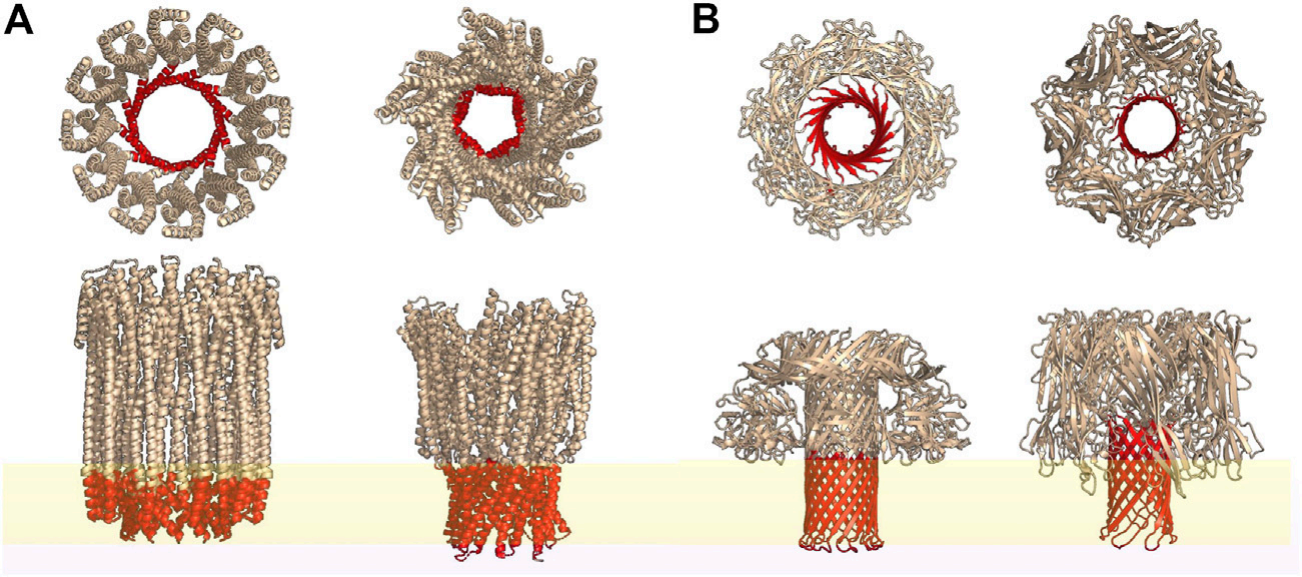
Top and side view representations of some typical *α*-PFTs **(A)** and *β*-PFTs **(B)** reconstructed from the PDB structures using PyMOL. Regions highlighted in red represent the transmembrane spanning secondary structures while yellow represents a lipid bilayer. From left: cytolysin A (PDB id: 6MRT); AhlB pore of AHL, from *Aeromonas hydrophila*; lysenin (PDB id: 5GAQ) and hemolysin (PDB id: 7AHL).

**FIGURE 2:**
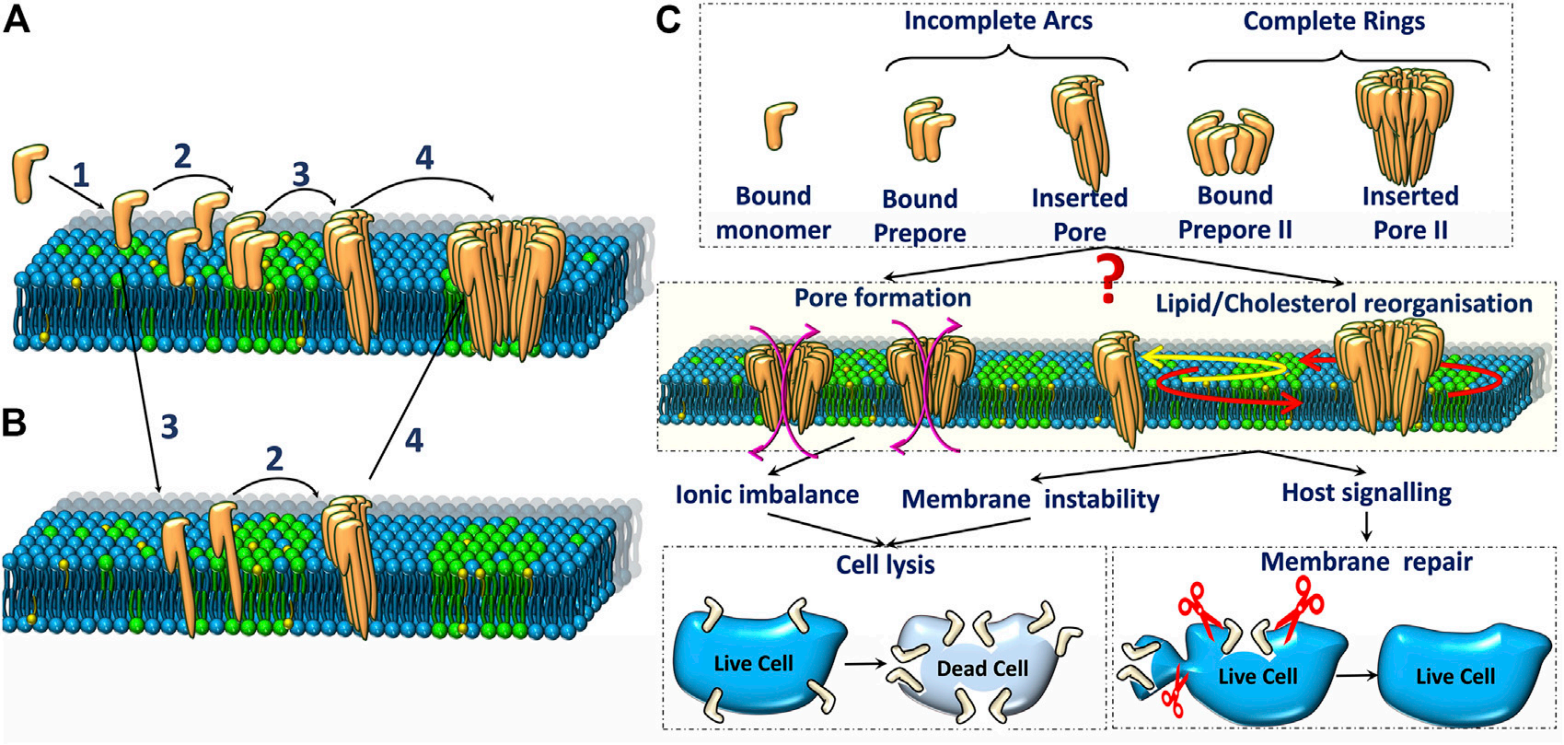
Schematic representation of two major pore formation pathways **(A**,**B)** of pore forming toxins. The various steps in **(A)** are 1. Binding of PFTs to the lipid bilayer, 2. Oligomerisation of monomers to form arc or ring-like prepore structures, 3. Membrane insertion, 4. Oligomerisation of inserted pores (observed in some cases). **(C)**. Various oligomeric states **(top)** and their influence on ionic imbalance and lipid reorganization **(middle)** triggers downstream repair processes which could result in either cell death or repair **(bottom)**.

Bacterial PFTs are secreted in the form of water soluble monomers in extracellular compartments (Gonzalez et al., 2008) or in extracellular vesicles that rupture upon interaction with host cells (Coelho et al., 2019). PFTs are broadly classified as *α*- and *β*-toxins based on the secondary structure of the membrane inserted motifs that constitute the transmembrane pores as illustrated in Figures 1A,B respectively. These differences in secondary structure influence the pore structure, protein-lipid interactions and pore formation pathways. Depending on the PFT, membrane binding is activated by specific receptors present on the mammalian cell membrane (Dal Peraro and van der Goot, 2016). These receptors could be specific lipids, cholesterol or proteins depending on the toxin. For example, lysenin, a typical bacterial *β*-PFT, targets sphingomyelin present on the rafts/nanodomains of the host cell membrane (Yilmaz et al., 2018) and cholesterol dependent cytolysins (CDCs) require cholesterol for pore formation. Although cholesterol enhances ClyA pore formation, lysis can take place in the absence of cholesterol as well. Upon binding to the target membrane, PFT monomers either oligomerize (Figure 2A) or insert into the host cell membrane (Figure 2B) forming transmembrane pores leading to cell lysis. The cell has evolved several defense mechansims to mitigate infections and repair pathways are triggered in order to recover membrane and cellular integrity (Etxaniz et al., 2018). The repair processes involve active or passive membrane remodeling events such as exocytosis and endocytosis in order to rid the membrane of the damaged toxin associated sites on the plasma membrane (Husmann et al., 2009; Keyel et al., 2011; Atanassoff et al., 2014). These processes which involve large membrane deformations are intrinsically connected to the underlying membrane fluidity.

The dynamics of lipids on the plasma membrane can be studied using a variety of different techniques ranging from X-Ray and neutron scattering methods to widely used optical microscopy techniques with variable resolution. Our earlier studies have revealed a strong connection between lipid dynamics measured using confocal and superresolution stimulated emission depletion (STED) fluorescence correlation spectroscopy (FCS) and the pore formation efficacy dependent on the specific phases present in the lipid membrane (Sarangi et al., 2016a; Sarangi and Basu, 2018; Ponmalar et al., 2019). Recent experiments with biomembranes exposed to typical *α*- and *β*-PFTs illustrate the intricate coupling of the lipid dynamics to membrane reorganization and pore formation (Sarangi et al., 2016b; Ponmalar et al., 2019).

At the molecular level, molecular dynamics (MD) simulations have provided several novel insights into local structure and dynamic reorganization that occurs during pore formation (Cheerla and Ayappa, 2020; Desikan et al., 2020; Varadarajan et al., 2020). MD simulations of PFT pore assemblies are computationally challenging due to the large extracellular regions. Simulations at different levels of coarse graining that reduce the degrees of freedom and hence the computational overhead have been carried out in our laboratory (Desikan et al., 2017; Sathyanarayana et al., 2017; Sathyanarayana et al., 2018; Cheerla and Ayappa, 2020; Desikan et al., 2020; Varadarajan et al., 2020) to shed light on the molecular mechanisms of pore formation and assembly. MD simulation methodologies that include all atom simulations with explicit solvent, coarse grained MARTINI models as well as structure based models to study PFTs have been recently reviewed by Desikan et al. (2021).

In this review, we will focus on results collated in our laboratory which provide insights into dynamics and lipid reorganization that occur during various stages of pore formation by two archetypal PFTs, cytolysin A (ClyA), an *α*-toxin and listeriolysin O (LLO), a *β*-toxin. We summarize key findings which probe the manner in which lipid dynamics is altered, using fluorescence microscopic techniques such as confocal and STED imaging, FCS, Förster Resonance Energy Transfer (FRET) and MD simulations ranging from all-atom to coarse grained methods. Since lipid dynamics is the primary focus, we briefly review various models that are commonly used to study lipid dynamics in different regimes drawing connections to the observations made in the presence of PFTs. Additionally, we briefly cover techniques such as atomic force microscopy (AFM) and neutron scattering techniques to probe lipid dynamics and reorganization in the presence of PFTs. Our review provides a collective view of PFT mediated plasma membrane disruption and lipid reorganization induced by changes in structure and local composition. The collective insights from these techniques link various stages of pore formation to protein induced lipid membrane response. We illustrate that probing lipid dynamics allows one to differentiate between *α*-PFTs and *β*-PFTs and their unique action on biomembranes. The review provides a current view on biomembrane response towards PFT attack and its implications in plasma membrane repair and cellular signaling mechanisms.

## 2 Pore Formation and Membrane Re-Organisation

The mechanism of PFT pore formation has been widely studied over decades (Dal Peraro and van der Goot, 2016). Pore formation involves three major steps: membrane binding, oligomerisation and insertion to form the transmembrane pore. These steps may be accompanied by a conformational change and the precise sequence of these different steps is only partially understood for certain classes of PFTs. As depicted in the schematic representation of the pore formation (Figures 2A,B), the initial binding of PFTs to the cell membrane can be followed by either oligomerisation or direct membrane insertion (Gonzalez et al., 2008). A typical *β*-PFT oligomerises into a prepore complex prior to membrane insertion (Figure 2C) driven by a conformational change to form either a membrane inserted arc or a complete ring inserted *β* barrel (Figure 1B). In the growing pore model, membrane insertion precedes oligomerization (Figure 2B). *α*-PFTs such as ClyA are thought to follow both pathways (Giri Rao et al., 2016; Sathyanarayana et al., 2020). The membrane inserted oligomers can form complete ring-like structures or incomplete arc-like structures. Interestingly, arcs and rings have been observed in both *α*-PFTs (Agrawal et al., 2017; Peng et al., 2019) and *β*-PFTs (Ruan et al., 2016). The different pathways that accompany pore formation give rise to unique protein-lipid interactions that have a direct influence on the underlying lipid dynamics. Hence the extent of disruption to membrane lipids from a prepore intermediate is likely to be different from that in a membrane inserted pore state. Further the extent of lipid perturbation is dependent on both the size of the pore complex, the secondary structure of the membrane inserted motifs as well as the topological variations of the pore complex, differentiated primarily by arcs or ring-like states (Figure 2C). In the current review, we focus on biomembrane dynamics during interactions of two widely studied *α*- and *β*-PFTs - ClyA and LLO, respectively. In addition, we include literature on other PFTs for which biomembrane dynamics have been studied. The pore formation pathway for these two classes for PFTs are briefly discussed next.

### 2.1 *β*- PFT: Listeriolysin O

The cholesterol-dependent cytolysins (CDCs) are *β*-toxins mostly produced by Gram-positive bacteria such as *Bacillus, Clostridium, Streptococcus and Listeria* (Tweten, 2005). CDCs can also be produced by certain Gram-negative strains (Hotze et al., 2013) as well. CDCs forms the largest (30–50 nm) transmembrane pore channels among the PFT family of proteins. Listeriolysin O (LLO) secreted by *Listeria monocytogenes*, a Gram-positive bacterium helps in the escape of *Listeria* from the lysosomes after the bacterium is endocytosed into the target cells through a phagocytic-like mechanism (Hamon et al., 2012). LLO is implicated in food poisoning related infections which causes listeriosis, fatal to immune-compromised individuals (Radoshevich and Cossart, 2018).

**FIGURE 3:**
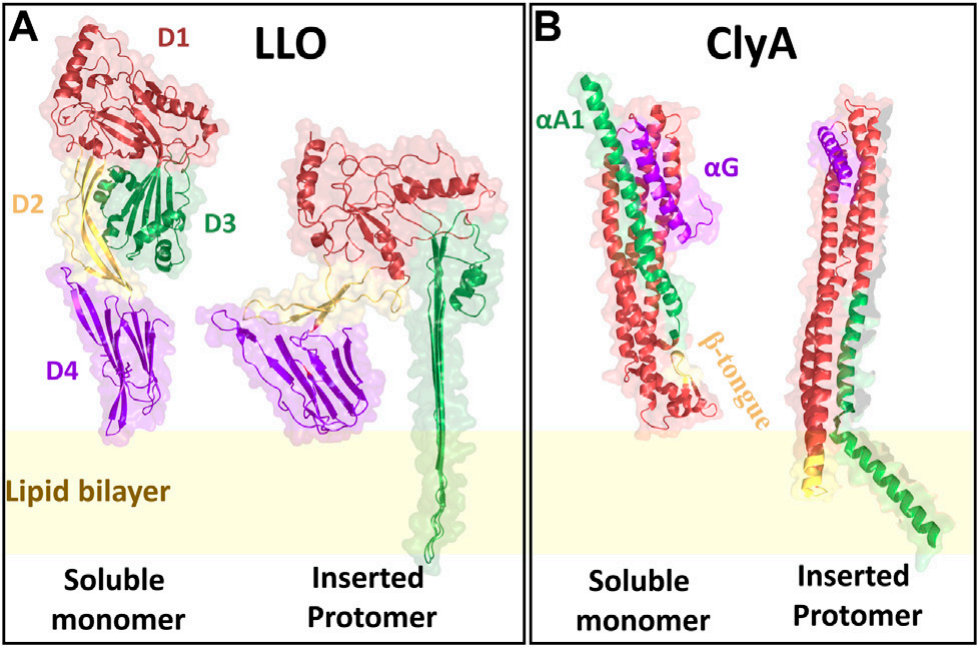
The crystal structures of LLO and ClyA monomers in their soluble as well as inserted states. LLO is structurally designated with four domains D1 (red), D2 (yellow), D3 (green) and D4 (purple). ClyA has three major motifs that play a significant role during pore formation: *β* tongue (yellow), C-terminal *αG* motif (purple) and N-terminal *αA*1 motif (green). The crystal structures are reconstructed from the PDB files using PyMOL. Since, the LLO inserted state crystal structure is not yet elucidated, the structure of Pneumolysin was used as to create the structure. For LLO monomer PDB id: 4CDB; Pneumolysin inserted state PDB id: 5LY6; ClyA soluble monomer PDB id: 1QOY; ClyA inserted state PDB id: 6MRT.

The process of pore formation by LLO is initiated by membrane binding of the D4 sub units (Figure 3A) of individual monomers to their membrane receptors, in this case cholesterol. Membrane bound monomers then oligomerize to form arcs and ring-like structures (Figure 2). Once the structures are formed, they undergo conformational changes to form membrane inserted arcs or *β* barrels to create functional pores (Bavdek et al., 2007) usually made up of ∼30–50 monomers (Bavdek et al., 2012). Recent studies using high resolution cryo-electron microscopy (cryo-EM) (van Pee et al., 2017) and high speed AFM (Ruan et al., 2016) of pore formation by CDC PFTs on model membranes have revealed the kinetics of pore formation including existence of prepore and pore states and the presence of a distribution of oligomeric states resembling arcs and rings (Podobnik et al., 2015; Ruan et al., 2016; Christie et al., 2018).

### 2.2 *α*- PFT: Cytolysin A

Cytolysin A (ClyA), a cytolytic *α*-PFT is typically secreted by certain virulent strains of *E. coli* (Ludwig et al., 2004) as well as *Salmonella enterica* (Oscarsson et al., 2002). ClyA is expressed as water soluble monomers and the crystal structure reveals the formation of a dodecameric pore assembly (Mueller et al., 2009). Pore formation is driven by the insertion of the hydrophobic *β* tongue (Figure 3B) into the membrane, accompanied by a large conformational change involving the swinging out of the N-terminus from the bundles of helices to form the membrane inserted pore state (Benke et al., 2015; Giri Rao et al., 2016; Desikan et al., 2017). Although the pore forming pathways for ClyA have been extensively investigated (Roderer and Glockshuber, 2017; Sathyanarayana et al., 2020) whether pore formation occurs via a prepore or growing pore model is a matter of debate. Evidence for partially inserted oligomeric states have been confirmed in MD simulations (Desikan et al., 2017) and implicated in leakage kinetic (Agrawal et al., 2017) models as well as CryoEM studies (Peng et al., 2019). The dual role played by cholesterol in assisting membrane insertion of the N-termini and stabilizing the pore state has been recently elucidated (Sathyanarayana et al., 2018; Peng et al., 2019).

## 3 Biomembrane Dynamics

The plasma membrane predominantly consists of lipids, sugars and transmembrane proteins which coexist to create a functionally complex 2D environment with a dynamic interplay of protein-lipid, lipid-lipid interactions and transport channels. The natural fluidic state of the plasma membrane is exploited by the cell for various cellular functions (Dowhan and Bogdanov, 2002). The inherent multicomponent nature gives rise to a dynamically and compositional heterogeneous landscape emerging as nano or microdomains in the plasma membrane. One way to assess the extent of this heterogeneity is to probe the dynamics of lipids and proteins on the plasma membrane as reflected in a measured diffusion coefficient. In this section after a brief overview of the basic diffusion regimes we discuss the role played by MD simulations and a host of experimental techniques, especially those based on fluorescence microscopy, that have been used to probe the dynamics of lipids at different length and time scales. In keeping with the focus of the review, we restrict our attention to diffusion of lipids.

### 3.1 Diffusion Laws

The diffusion coefficient, *D* of a given particle in 2D is evaluated using the Einstein relation,

$$D=\underset{t\to \infty }{\lim}\frac{1}{4t}\left\langle |r\left(t\right)-r\left(0\right){|}^{2}\right\rangle$$ (1)

where |**r**(*t*) − **r**(0)|^2^ = Δ**r** ^2^(*t*), is the mean squared displacement (MSD) of the particle at time *t*. The angular brackets in general represent averages over the number of particles as well as the number of different time origins being sampled in a specific region. Lipid diffusion in biomembranes typically occurs in a heterogeneous environment and several diffusive regimes have been observed using single particle tracking (SPT) experiments as well as with molecular dynamics (MD) simulations. To discern between Brownian and non-Brownian or anomalous regimes one examines the scaling of the mean squared displacement at long times using,

$$\Delta r^{2}\left(t\right)∼t^{\alpha }$$ (2)

where, the exponent *α* is customarily referred to as the anomaly parameter. When *α* = 1, the dynamics is diffusive or Brownian and the MSD versus time data yields the particle diffusivity, *D* (Eq. 1). However when *α* ≠ 1, diffusion is anomalous, with *α* < 1 referred to as a sub-diffusive regime and *α* > 1 knows as the super diffusive regime (Metzler et al., 2016). The anomalous nature of lipid diffusion is typically reported to be sub-diffusive with *α* < 1 when the lipids are crowded by large external molecules such as proteins or peptides (Jeon et al., 2016).

Anomalous diffusion has been widely observed in lipid dynamics of multicomponent membranes induced by compositional heterogeneity as well as the presence of transmembrane proteins (Niemelä et al., 2010; Jeon et al., 2016; Javanainen et al., 2017). Thus the manner in which the free particle motion is modulated in these different environments have been referred to as either trapped or hop diffusion (Sarangi et al., 2018) to primarily differentiate between the underlying restrictions to particle motion. The lipid diffusion in the domains will be usually slower in comparison to the surrounding environment (Simons and Vaz, 2004; Lingwood and Simons, 2010).

Lipid diffusion coefficients ideally depends on various physical parameters such as temperature, area per lipid as well as the chemistry of the specific lipid. Among these parameters, dependence on the area per lipid has been quantified using free area models (Almeida et al., 1992; Almeida et al., 2005). These free area models are an extension of the free volume models described earlier by Cohen and Turnbull (1959). In the free area models by Almeida et al. (1992), the relationship between the lipid diffusivity, *D* and the lipid free area, *a*_*f*_ is given by,

$$D=D_{0}e^{-\frac{a_{o}}{a_{f}}}$$ (3)

where, *D*_0_ is the reference lipid diffusion coefficient, *a*_*f*_ is the free area per lipid, *a*_*o*_ is the minimum area per lipid above which lipid diffusion occurs.

In what follows, we briefly present various techniques which allows the estimation of lipid diffusivities and other relevant biomembrane dynamical parameters. We begin by first discussing how *D* as well as protein induced heterogeneities can be estimated using MD simulations for model biomembranes. Subsequently several experimental techniques at various spatiotemporal scales that are used to probe lipid dynamics are discussed.

### 3.2 Dynamics From Molecular Dynamics Simulations

**FIGURE 4:**
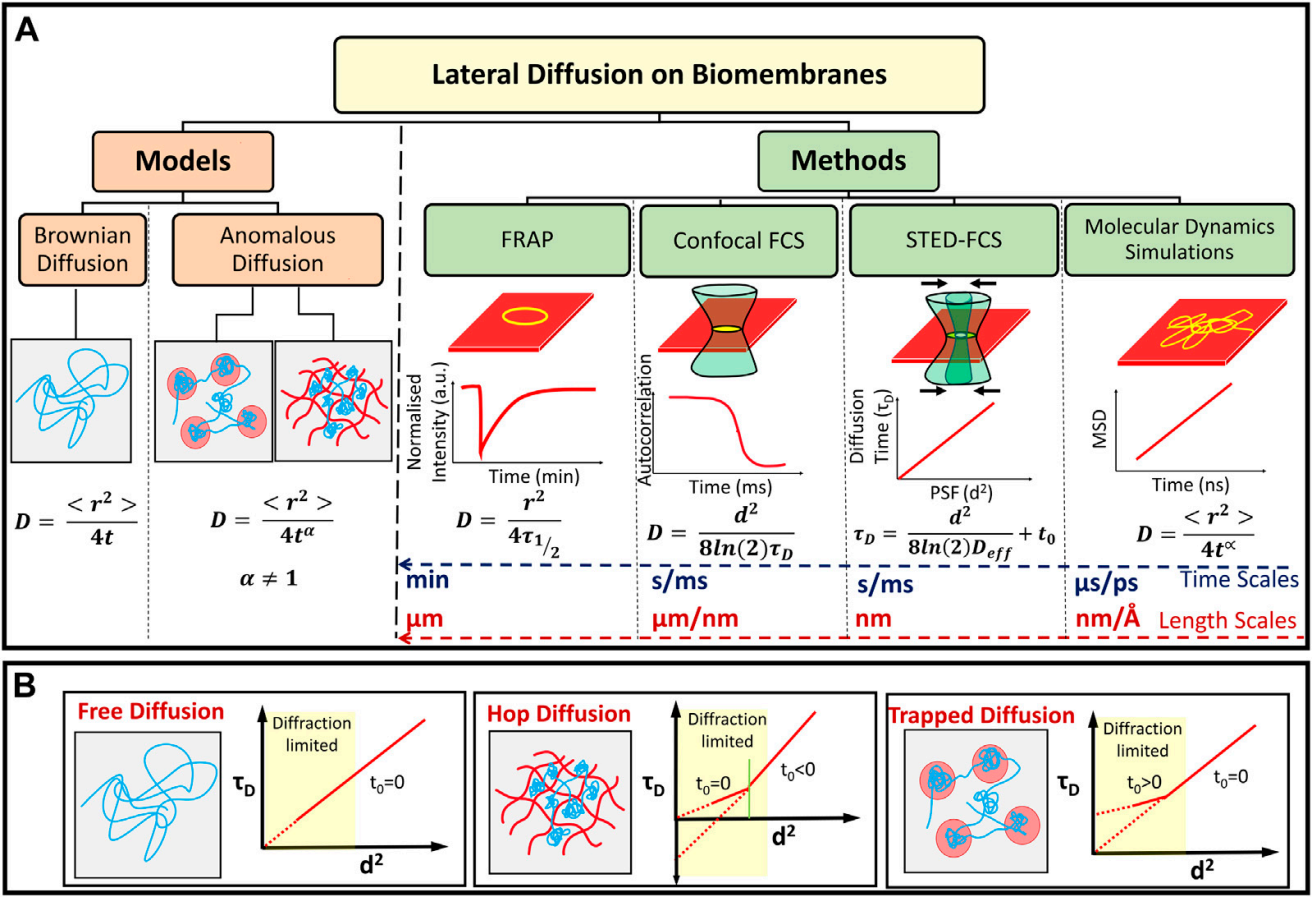
**(A)** Models of lipid diffusion in biomembranes and methods used to study molecular diffusion at different length and time scales. Diffusion can be classified as anomalous or Brownian depending on the value of the parameter *α*. The typical temporal and spatial resolution for probing membrane lipid diffusion using various experimental techniques as well as MD simulations are indicated. **(B)** Schematic representation of different τ_D_ vs d^2^ curves attributed to free, hop and trapped diffusion mechanisms observed on lipid cell membranes (Lenne et al., 2006; Winkler et al., 2018).

Molecular dynamics (MD) simulations have been extensively used to study dynamics of lipids in phospholipid bilayers ranging in complexity to encompass single and multicomponent bilayers (Marrink et al., 2009), bilayers with transmembrane proteins (Stansfeld and Sansom, 2011) and more recently to bilayers with the larger PFT oligomeric complexes (Aksimentiev and Schulten, 2005; Desikan et al., 2017; Sathyanarayana et al., 2018; Vögele et al., 2019; Cheerla and Ayappa, 2020; Desikan et al., 2020). MD simulations involve integrating the equations of motion to evolve positions and velocities of particles in different ensembles as a function of time, and the accuracy of the dynamics are a direct function of the intra and intermolecular potentials used to describe the forces between the molecules. In addition to static structural characterization involving the density variations, and lipid order parameters, several dynamical quantities such as the MSD (Eq. 1), time correlation and scattering functions can be obtained from the particle trajectories (Varadarajan et al., 2020) as illustrated in Figure 4B. The transition of the dynamics from sub-diffusive to diffusive regimes (Javanainen et al., 2013) can also be evaluated provided the simulations have been carried out for sufficiently long times (>1 µs).

Since the presence of proteins and lipids in an inherently multicomponent membrane leads to dynamical heterogeneities a measure of the local spatial variation in particle displacements can be monitored using a particle “mobility,” *μ*, defined as

$$\mu_{i}=\frac{\left|r_{i}\left(t+\Delta t\right)-r_{i}\left(t\right)\right|}{\Delta t}$$ (4)

where *μ*_*i*_ is the mobility of the *ith* lipid molecule, *r*_*i*_(*t*) and *r*_*i*_(*t* + Δ*t*) are the coordinates of the center-of-mass (COM) of the *ith* lipid molecule in the membrane plane at time *t* and time *t* + Δ*t*, respectively. Two dimensional maps of the mobility or the displacements of the particles with reference to the original position provides a spatial measure of the extent of dynamic variations at the single particle level present in the membrane. MD simulations have also been extensively used to study binding sites for cholesterol and lipids on the proteins (Prasanna et al., 2014).

### 3.3 Experimental Methods to Study Lipid Diffusion

Experiments can be carried out to measure the lateral diffusion of lipids and other molecules on the membrane (Figure 4A). These methods typically image and collect data (lateral mobility) in micron-sized or nano-sized regions of the membrane in order to measure diffusion in specific regions of single cells or vesicles (Chen et al., 2006). The methods are primarily based on the fluorescence emission from dye molecules which are embedded in the membrane or are tagged onto lipids which can homogeneously mix with unlabelled membrane lipids. The concentration of such fluorescently tagged molecules are typically smaller than that of the molecules of interest (lipids) with the actual number depending on the technique being used.

#### 3.3.1 Fluorescence Recovery After Photo Bleaching

Fluorescence recovery after photo bleaching (FRAP) is one of the techniques that exploits time-lapse imaging coupled with photo-bleaching to estimate the diffusion constant of lipid in supported lipid membrane (Kang et al., 2012). In FRAP, we image lipids with higher dye concentration of a molar ratio from 0.01 to 0.1 (Day et al., 2012). Usually, a small circular portion of the lipid membrane of size 5–10 μm is bleached with a high intensity laser. During imaging, one observes the intensity recovering in the bleached area (Chen et al., 2006). The recovery of the fluorescence intensity inside the bleached area is recorded as a function of time and then fitted to an exponential function to obtain a characteristic time, *τ*_1/2_, which is defined as the time required to reach 50% of complete recovery. Assuming that the fluorescence recovery occurs only by 2D diffusion, the diffusion coefficient, *D*, is given by,

$$D=\frac{r^{2}}{4\tau_{1/2}}$$ (5)

where, *r* is the radius of the bleached area.

#### 3.3.2 Single Particle Tracking

Single particle tracking (SPT) is a widely used technique to measure the diffusion of any fluorescent molecule. Theoretically, molecular dynamics simulations employ this principle to measure the lipid and cholesterol diffusion coefficients. Experimentally, SPT is typically used when the diffusion is slow enough to be captured using the detectors in a total internal reflection fluorescence (TIRF) microscope (Shen et al., 2017). Although the basic principle of SPT can be extended to confocal and STED imaging systems, TIRF provides faster time-lapse imaging due to the use of single shot wide field imaging methodology based on charge couple device (CCD) camera rather than photon counting detectors such as photo-multiplier tubes or avalanche photo diode detectors (Manzo and Garcia-Parajo, 2015). The rapid image capture by the CCD camera is facilitated due to its ability to image an array of pixels simultaneously whereas, PMTs and APDs require sequential pixel-by-pixel imaging. It requires very few labelled molecules to be present in the system for identification and tracking to be efficient. The trajectories of any fluorescent molecule, lipids or proteins are recorded continuously using computer-enhanced video capture technique (Manzo and Garcia-Parajo, 2015).

These trajectories of individual molecules are then analysed using standard algorithms (Shen et al., 2017) to localise individual particles and further identify different types of motion exhibited by the molecules using neural network models (Kowalek et al., 2019). Averaged trajectories yield < *r* ^2^ > as a function of lag times which can be fitted to standard equations such as Eq. 2, to identify the changes in motion that the lipids (Schoch et al., 2018) and proteins undergoes on the lipid bilayer. A major advantage of SPT is the ability to resolve the different modes of motion that an individual molecule undergoes. One of the major results that can be interpreted from this technique is that lateral motion of a single molecule in the membrane is not limited to pure diffusion (Slator et al., 2015). When the population of diffusing molecules are heterogeneous in nature, which is to be expected in case of multi-component lipid bilayers interacting with external molecules, SPT is advantageous over spatially averaged techniques like FRAP (Saxton and Jacobson, 1997).

#### 3.3.3 Fluorescence Correlation Spectroscopy

Fluorescence correlation spectroscopy (FCS) is a widely used method for measuring translational molecular diffusion on biomembranes by quantifying the fluorescence intensity fluctuations observed inside a confocal beam (Thompson, 2002). In this method, the recorded fluctuations are temporally autocorrelated to reveal information about the concentration and dynamics of the fluorescent species (Elson and Magde, 1974). FCS has been the subject of many reviews (Hess et al., 2002; Tian et al., 2011) and texts (Rigler and Elson, 2012).

In the case of 2D free Brownian diffusion typically observed in membranes, the temporal autocorrelation function is given by,

$$G\left(\tau \right)=\frac{1}{N}{\left(1+\frac{t}{\tau_{D}}\right)}^{-1}+c$$ (6)

where, *N* is the number of fluorescent molecules in the beam at any time point *t*, *τ*_*D*_ is the characteristic diffusion time taken by the molecule to move across the beam, and *c* is a constant that corrects for background noise. In real systems, the type of diffusion is usually unknown and if the molecule is suspected to have anomalous diffusive behavior, the parameter *α* is usually introduced. Deviations of *α* from unity provide the first signatures of anomalous diffusion. Additionally, some fluorophores have characteristic triplet state which modifies the equation to,

$$G\left(t\right)=\frac{1}{N}\frac{\left(1-\theta_{t}+\theta_{t}e^{-\frac{t}{\tau_{t}}}\right)}{1-\theta_{t}}\frac{\rho }{{\left(1+\frac{t}{\tau_{D}}\right)}^{\alpha }}+c$$ (7)

where, *θ*_*t*_ is the fraction that is in the triplet state, *τ*_*t*_ is the time duration for the molecule to remain in the triplet state and *ρ* is the fraction of diffusing molecules. In all situations, the diffusion coefficient, *D*, is calculated from the characteristic diffusion time *τ*_*D*_ using,

$$D=\frac{d^{2}}{8\tau_{D}ln\left(2\right)}$$ (8)

where, *d* corresponds to the radial dimension of the confocal beam given by Full-Width Half Maxima (FWHM) of the Gaussian beam profile.

#### 3.3.4 Nanoscale Dynamics by STED-FCS

Advent of superresolution techniques revolutionized the field of microscopy by making use of fluorophore photo physics. This opened up imaging regimes to a much smaller length scale than accessible with conventional light based methods, entering into a new nanoscopy regime by breaking the Abbes’ diffraction limit (Hell and Wichmann, 1994; Roobala et al., 2018; Sezgin et al., 2019). Recent advancements to these techniques allows 20 nm lateral resolution and 100 nm axial resolution thereby attaining 10–15 times enhanced resolution when compared to conventional optical microscopes. Super-resolution STED works along a similar principle to confocal imaging, with an additional depletion laser used to enable the high spatial resolutions (Hell and Wichmann, 1994).

As the STED depletion laser power is increased gradually, there exists an exponential relationship with the observed diameter, *d* of the point spread function (PSF) of the excitation beam given by,

$$d\approx \frac{\lambda }{2NA\sqrt{1+\frac{I}{I_{s}}}}$$ (9)

where, *λ* is the wavelength of the excitation laser, *NA* is the numerical aperture of the objective, *I* is the STED laser intensity and *I*_*s*_ is the saturation intensity which depends on the fluorescent molecule. When we couple this basic STED imaging setup, which essentially reduces the observation length scale *d* of Eq. 8 with a photon counting module, we can measure the diffusion coefficient of the fluorescent molecules at varying length scales (Mueller et al., 2013; Chelladurai et al., 2018; Sarangi et al., 2018) usually termed as STED-FCS. This was demonstrated on live cell membranes by Eggeling et al. (2009) to identify lipid dynamics at the nanoscale. 

Spot-variation FCS (sv-FCS) is a modified version of the standard FCS wherein the diffusion time τ_D_ is monitored as a function of *d^2^*, the PSF of the incident laser beam. Before the advent of STED, the confocal PSF was typically varied with the help of a diaphragm or a variable telescope and by the lateral extension of the laser excitation beam falling onto the back-aperture of the microscope objective (Wawrezinieck et al., 2005). However, with the advent of STED, coupling STED with FCS extended the spot variation FCS into the diffraction limited regime (Eggeling et al., 2009; Sezgin et al, 2017). Additionally, using optical nanoantenna based FCS, the sv-FCS was used to identify nanoscale heterogeneities on live cell membranes (Lenne et al., 2006; Winkler et al., 2018; Regmi et al, 2017). 

The relationship between diffusion coefficient with *τ*_*D*_ is similar to that given in Eq. 8. However in spot variation STED-FCS where *τ*_*D*_ is evaluated at different values of *d*, Eq. 8 can be modified as (Ng et al., 2015),

$$\tau_{D}=\frac{d^{2}}{8D_{e\mathrm{ff}}ln\left(2\right)}+t_{0}$$ (10)

to account for a non-zero intercept (*t*_0_) in the FCS diffusion law (Eq. 8) and *D*_*eff*_ is an effective diffusion coefficient. *t*_0_ = 0 for free Brownian diffusion where *D*_*eff*_ = *D*, *t*_0_ > 0 corresponds to diffusion confined in domains while *t*_0_ < 0 has been attributed to diffusion in mesh-work hindered or gel-like environments (Lenne et al., 2006; Sarangi et al., 2018; Veerapathiran and Wohland, 2018) as illustrated in Figure 4B. Furthermore, from Eq. 10, *D*_*eff*_ can be obtained from the *τ*_*D*_ versus *d* ^2^ data. When *t*_0_ ≠ 0, the interpretation of *D*_*eff*_ is not straightforward, although fitting of individual FCS correlation curves for a given *d* ^2^, to Eq. 6 or Eq. 7 can still lead to an estimation of diffusion coefficient using Eq. 8. In situations for *α* < 1 we refer to *D* as *D*_*app*_. For the special case, when *t*_0_ < 0, the size of the nanodomains *ω* is extracted (Favard et al., 2011; Sarangi et al., 2017; Sarangi and Basu, 2019) by setting *τ*_*D*_ = 0 in Eq. 10 to obtain,

$$\omega =-\sqrt{8D_{e\mathrm{ff}}ln\left(2\right)|t_{0}|}.$$ (11)

### 3.4 Other Experimental Methods for Determination of Membrane Dynamics

Other than the above discussed classical methods to calculate the lipid lateral diffusion dynamics during PFT attack, certain standard methods are used to identify the biomembrane morphological and dynamical changes upon PFT attack. We have briefly discussed two major methods that are widely used in the PFT-lipid structural and dynamic studies.

#### 3.4.1 High Speed AFM

One of the scanning force microscopy techniques, atomic force microscopy (AFM) works on the principle that the force experience by a cantilever probe is related to the surface properties of the sample (Mingeot-Leclercq et al., 2008). Unlike the conventional AFM, high speed AFM can obtain images at the rate of 15–20 frames per second (Ando et al., 2001). This faster imaging technique has enabled one to study the dynamics of biomolecules at high resolutions using AFM. High speed AFM has been used to study the kinetics of pore formation along with nanoscale structural information during the membrane disruption process (Jiao et al., 2021). For enabling the high speed imaging, Ando et al. (2001) have developed a high-speed scanner free of resonant vibrations up to 60 kHz and during imaging, using small cantilevers with high resonance frequencies (450–650 kHz) and small spring constants (150–280 pN/nm). They also modified the classical deflection detector to objective-lens type detector and added several electronic devices of wide bandwidth.

#### 3.4.2 Neutron Scattering Studies

Another classical method for elucidating the structural and dynamic changes that happens during lipid protein interaction include neutron scattering techniques such as inelastic and quasielastic neutron scattering (Swenson et al., 2008; Wood and Zaccai, 2008; Armstrong et al., 2012) as well as neutron spin echo (Kumarage et al., 2021). The quasielastic neutron scattering data can be converted to the dynamic nature of lipid membranes based on scattering laws (Qian et al., 2020). Based on the broadening or narrowing of the quasielastic peaks, the order of the lipid bilayer can be extracted. Using quasielastic neutron scattering, Sharma et al. (2015) have reported that an antimicrobial peptide (AMP), melittin enhanced the lipid lateral diffusion in the absence of cholesterol whereas aurein, another AMP was observed to reduce lateral lipid mobility (Sharma and Qian, 2019). Kinnun et al. (2021) have recently reviewed in detail the advantages of using neutron scattering in determination of membrane dynamics as well as nanoscale membrane features. The main advantage of neutron scattering over confocal FCS is the use of label free techniques in the lipid- protein interactions.

#### 3.4.3. Interfermetric Scattering Microscopy

One of the recent label free techniques that has been used to measure protein diffusion involves detection and tracking of Rayleigh scattering observed from the proteins and lipids tagged with gold nanoparticles. Spindler et al. (2016) have emphasized that while high speed dynamic tracking of lipids and proteins are improved while using scattering labels such as gold or polymer nanoparticles, they also mention that unlabeled lipids and proteins can also be imaged using iSCAT provided the polarizability of the molecule of interest is sufficiently large. On comparing the use of iSCAT with STED-FCS on lipid membrane dynamics, Reina et al. (2018) have reported that the values of apparent diffusion coefficients obtained by from STED-FCS and iSCAT differed by a factor of 2–3 across the techniques, while relative differences in mobility between different species of lipid analogues considered were identical in both approaches.

## 4 Lipid Reorganisation and Dynamics During PFT Interaction: LLO and ClyA

In this section, we discuss the findings related to lipid membrane reorganization and dynamic modulations therein due to the presence LLO and ClyA on the membrane. The different pathways that accompany pore formation give rise to unique protein-lipid interactions that have a direct influence on the underlying lipid dynamics. Hence the extent of disruption to membrane lipids from a prepore intermediate is likely to be different from that in a membrane inserted pore state. Further the extent of lipid perturbation is dependent on both the secondary structure of the membrane inserted motifs of pore complex as well as the topological variations of the pore complex, differentiated primarily by arcs or ring-like states (Figure 2C). In what follows, we describe insights gained from experiments and MD simulations that broadly fall into two regimes. The confocal-FCS measurements probing length scales above 250 nm and the STED-FCS measurements which probe dynamics down to ∼50 nm as well as MD simulations which provide microscopic interpretations at smaller length and time scales (Figure 4A). We discuss and differentiate how specific lipid domains influence membrane binding and pore formation by *α*- and *β*-toxins. In addition the manner in which PFTs uniquely induce and create dynamical heterogeneities in an otherwise homogeneous lipid environment will be discussed. We will also discuss PFT concentration dependent lipid diffusion induced by interplay of lipid ejection and crowding to reveal changes in membrane bound oligomeric state populations of PFTs. Major lipids discussed in this review include 1,2-dioleoyl-*sn*-glycero-3-phosphocholine (DOPC), 1-palmitoyl-2-oleoyl-*sn*-glycero-3-phosphocholine (POPC), 1,2-dipalmitoyl-*sn*-glycero-3-phosphocholine (DPPC), 1,2-dimyristoyl-*sn*-glycero-3-phosphocholine (DMPC), sphingomyelin (SM), and cholesterol (Chol).

### 4.1 Biomembrane Dynamics in the Presence of LLO

The influence of LLO binding and pore formation on lipid lateral diffusion was explored using confocal FCS on supported lipid bilayers (SLBs) (Sarangi et al., 2016a; Sarangi et al., 2016b; Sarangi and Basu, 2018; Sarangi and Basu, 2019; Ilanila et al., 2021) and giant unilamellar vesicles (GUVs) (Ponmalar et al., 2019). In some cases, FRET has also been used to correlate the protein bound states with the observed lipid dynamics.

**FIGURE 5:**
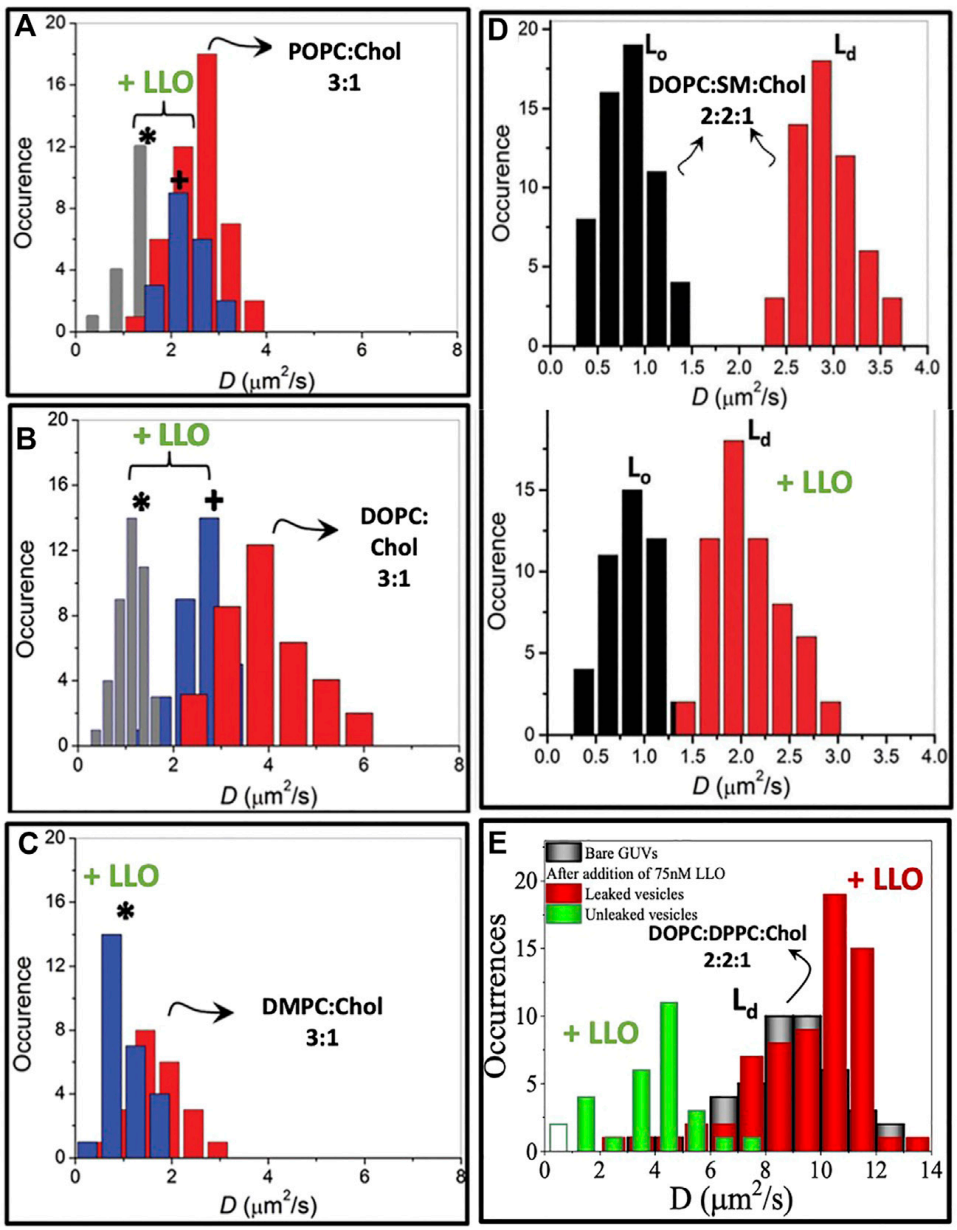
Lipid diffusion coefficients *D* measured from lipid bilayers of different compositions using confocal FCS. Panels **(A**–**C)** demonstrate hindered bimodal lipid diffusion observed on SLBs of compositions POPC, DOPC and DMPC with 25% cholesterol at high LLO concentrations of 86.2 nM. Measurements collected in the vicinity of the pore complex (*) and away from the pore complex (+). Adapted from Sarangi et al. (2016b). **(D)**. In a three component lipid bilayer comprised of DOPC:SM:Chol, the changes in the lipid diffusion upon LLO interaction is observed specifically on the L_*d*_ domain. Adapted from Sarangi and Basu (2018). **(E)**. Lipid *D* measured on L_*d*_ domain of DOPC:DPPC:Chol GUVs indicating bimodal distribution in leaked and unleaked vesicles. Reproduced from Ponmalar et al. (2019).

The first signatures of LLO induced lipid dynamical heterogeneities is observed in two component phospholipid-cholesterol bilayers. On experiments carried out with 25% cholesterol the single population of lipid diffusivities changes to two distinct populations of lipid diffusivities upon addition of LLO (Figure 5). This situation is observed for both POPC:Chol and DOPC:Chol membranes as illustrated in (Figures 5A,B). The slower moving lipid population (denoted by ∗) corresponds to regions in the vicinity of the pore complex. In addition, the nature of underlying membrane fluidity based on the lipid melting points influence the observed dynamic reorganization. Upon combining the results from confocal-FCS with AFM imaging, lower melting bilayers are prone to form LLO pores readily in comparison to the high melting bilayers. Hence the induced heterogeneity in DOPC:Chol (3:1) membranes is the greatest (Figure 5B). Thus we correlate the slow moving lipid population in the pore vicinity to the higher local cholesterol content and the faster moving lipid population arising from the cholesterol depleted regions away from the pore complex. We revisit this aspect later in the text. In contrast, for the high melting DMPC membrane, the perturbation as observed in the emerging lipid populations (Figure 5C) is the least, and pore formation is not observed in AFM images. Intermediate diffusivity modulations occur in the POPC:Chol membranes (Figure 5A). At higher cholesterol concentrations (50%) in two component phospholipid bilayers sub micron scale domains emerge giving rise to a heterogeneous lipid diffusivities in the absence of LLO, with both populations shifting to lower diffusivities upon LLO addition (Sarangi and Basu, 2019).

In three component DOPC:SM:Chol (2:2:1) bilayers where liquid ordered, L_*o*_ and liquid disordered, L_*d*_ domains are observed, monitoring the lipid diffusivity changes in these different domains reveals several insights into LLO binding and pore formation. The diffusivity in the L_*o*_ phase is relatively unchanged, however the diffusivity of lipids in the L_*d*_ domains shows a distinct decrease (Figure 5D). This shows that LLO preferentially binds to the L_*d*_ phase where cholesterol is more readily transported due to higher lipid mobility when compared with the less mobile and rigid L_*o*_ phase.

#### 4.1.1 Oligomeric Intermediates of LLO on Lipid Dynamics

**FIGURE 6:**
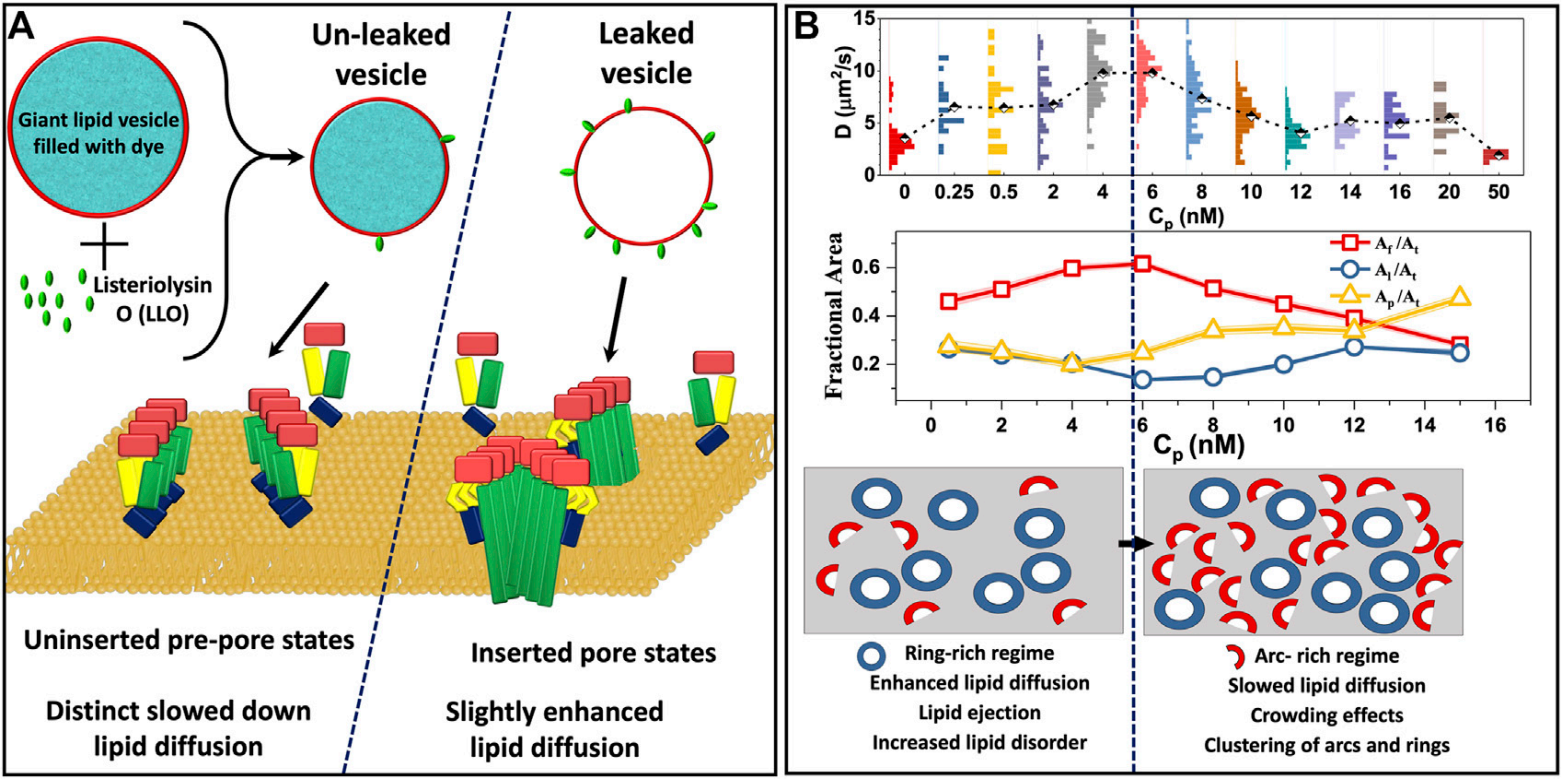
**(A)**. Schematic representation of altered lipid dynamics on GUVs identified based on the leakage and FRET experiments. Uninserted prepores result in slowed lipid diffusion whereas inserted pores are found to enhance lipid diffusion (Ponmalar et al., 2019). **(B)**. Lipid diffusion coefficients measured as a function of LLO concentration, *C*_*p*_ on supported lipid bilayer experiments **(top)** and corresponding changes in the lipid free area **(middle)** derived using Eq. 3. *A*_*l*_, *A*_*f*_, *A*_*p*_ and *A*_*t*_ represent the area occupied by the lipid, free area in the membrane, area of protein and the total membrane area respectively. Using a two state model (Ilanila et al., 2021) where LLO oligomers are present as either rings or arcs, the increasing lipid diffusivity at low *C*_*p*_ corresponds to a regime dominated by rings, and the decreasing diffusivity at higher *C*_*p*_ corresponds to an arc-rich crowded regime **(bottom)**. **(B)** Adapted from Ilanila et al. (2021).

In order to identify a link between the different oligomeric states observed during LLO pore formation and the induced changes in the underlying lipid dynamics, Ponmalar et al. (2019) have analyzed lipid diffusion data collected from GUV leakage experiments combined with FRET to correlate the oligomeric states with leakage. Time dependent lipid dynamics measurements are carried out on GUVs made up of DOPC:DPPC:Chol (2:2:1) filled with fluorescent dye cyanine-3–N-hydroxysuccinimido (Cy3-NHS ester) (Ponmalar et al., 2019). Dye leakage from Cy3 encapsulated within the giant unilamellar vesicles (GUVs) was used as an indicator of pore formation (Figure 6A). A distinct lowering of lipid diffusivities when compared with bare vesicles was observed in unleaked vesicles (Figure 5E). In contrast, the lipid diffusivities were found to increase in the leaked vesicles indicating the increased membrane fluidity induced due to LLO insertion and pore formation. The lipid dye, Atto647N-DMPE, used in FCS experiments partitions into the cholesterol poor L_*d*_ phase where LLO preferentially binds. FRET efficiency measurements between the protein and lipid provided additional evidence of membrane bound states associated with unleaked vesicles and membrane inserted states associated with the leaked vesicles to confirm the non-monotonic dependence on lipid diffusivities in time-lapse FCS measurements. In all cases, the anomaly parameter *α* (Eq. 7) in the FCS correlation curves was unity indicative of free lipid diffusion in these tethered GUV ensemble experiments.

In order to study the influence of LLO pore formation on lipid dynamics in a more controlled environment, FSC experiments were carried out on SLBs over a range of LLO concentrations, C_*p*_ (Ilanila et al., 2021) in a homogeneous lipid bilayer system (DOPC:Chol:3:1) as well as in the L_*d*_ domain of a phase separated, DOPC:DPPC:Chol:2:2:1, bilayers. Lipid diffusion was observed to have an initial enhancement followed by a gradual decline upon increasing *C*_*p*_ (Figure 6B). Combined with FRET experiments and a free area model (Eq. 3) for diffusion, the study proposed a two state model that revealed a direct correlation with different membrane inserted states and the non-monotonic variation in the lipid diffusivities observed as a function of protein concentration. The two state model assumes that the membrane inserted oligomeric states can exist as either arcs or rings. In the two state model the amount of protein on the lipid bilayer is a linear combination of arcs and rings given by,

$$A_{p}=\alpha_{A}A_{a}+\beta_{R}A_{r}$$ (12)

where *A*_*p*_ is the total area of proteins on the bilayer, *A*_*a*_ is the area occupied by arcs, *α*_*A*_ is the fraction of arcs, *β*_*R*_ is the fraction of rings and *A*_*r*_ is the area occupied by the rings. Using this two state model, the enhanced lipid mobility observed at lower protein concentrations (2 nM < C_*p*_ < 6 nM) was linked to the formation of ring-like pores where lipid ejection was dominant, increased free area per lipid and higher lipid disorder due to membrane insertion. At higher concentrations (C_*p*_ > 6 nM) the membrane has a higher population of arc-like pore structures giving rise to a more crowded environment, resulting in a decrease in lipid diffusivities. The interesting observation was the existence of a cross-over protein concentration at which ring-like pore formation saturates and lipids increasingly feel the effect of crowding due to the presence of arc-like pores on the membrane. These different diffusive regimes that emerge as a function of *C*_*p*_ are illustrated in Figure 6B.

#### 4.1.2 Nanoscale Lipid Dynamics and Re-Organisation due to Crowding of LLO

The resolution of confocal FCS experiments carried out on GUVs and SLBs reveal information above a length scales of ∼240 nm, however, the lipid reorganization that occur around pores or pore clusters at smaller length scales is unresolved. Higher resolutions to resolve lipid dynamics below the diffraction limit can be achieved using spot-variation measurements can thus be captured using STED-FCS measurements. We summarize these results next. As explained in Figure 4, using spot-variation STED-FCS measurements one obtains the value of the characteristic diffusion behaviour of lipid and/or protein in a spot variation manner below the diffraction limit, which is otherwise difficult to achieve in any conventional optical microscopic method.

**FIGURE 7:**
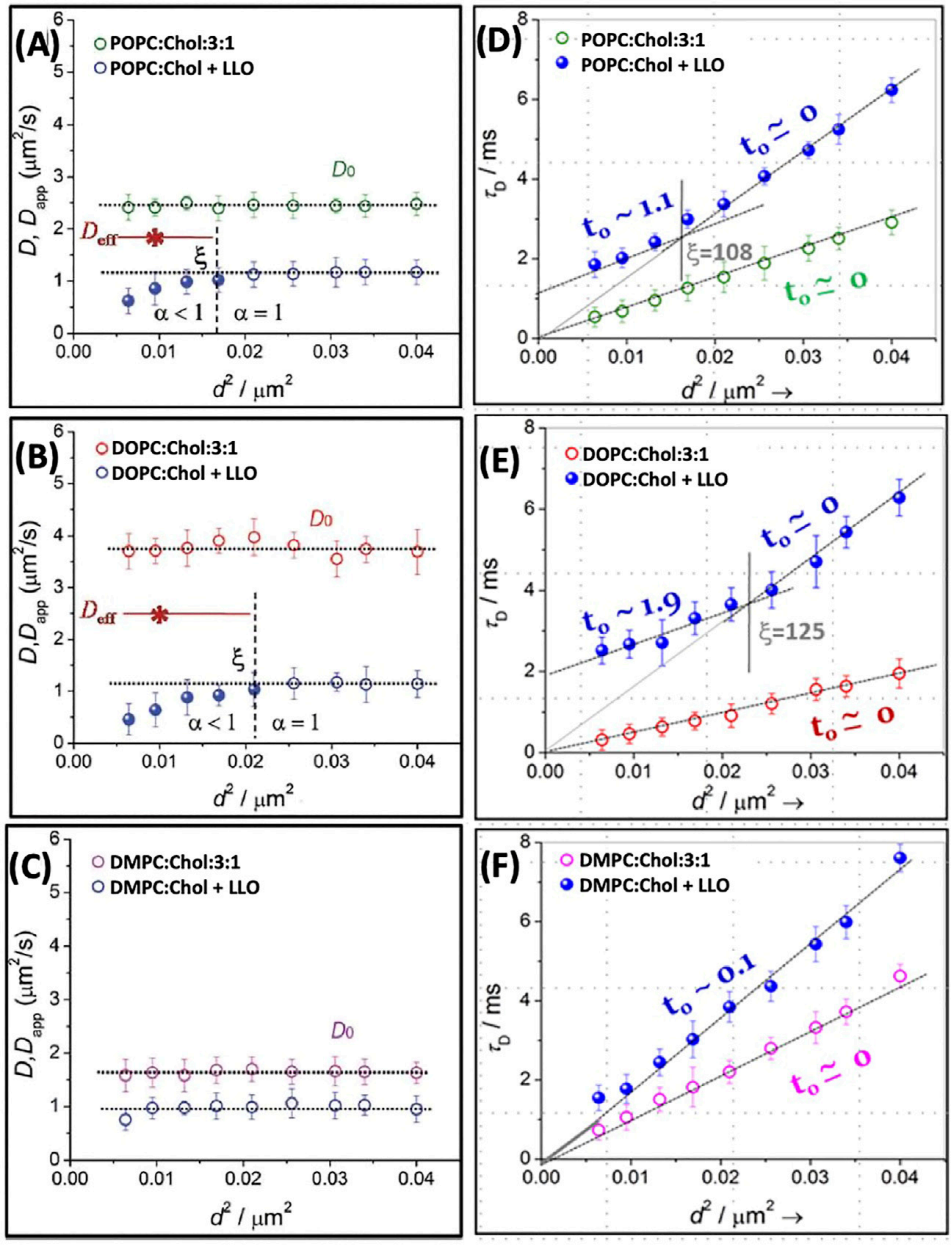
The diffusion coefficients *D* and *D*_*app*_ plotted as a function of *d* ^2^ for POPC **(A)**, DOPC **(B)** and DMPC **(C)** with 25% Chol. We have re plotted the results in the form of *τ*_*D*_ vs. *d* ^2^ **(**Panels **D**–**F)**. The solid lines in the respective panels are the linear fit using Eq. 10 and the dotted lines are the extrapolated line to show the intercept. The vertical lines, in respective panels, demarcates the crossover length scale, *ξ*, separating different dynamical regimes whether Brownian or non-Brownian diffusion. Adapted from Sarangi et al. (2016b).

Using STED-FCS, Sarangi et al. (2016b) report diffusion at length scales <200 nm in POPC, DOPC and DMPC bilayers with 25% cholesterol. In the absence of LLO, free Brownian diffusion is observed, as reflected in a constant diffusivity value with respect to the square of the spot size, *d* ^2^ (Figures 7A–C). In contrast, upon LLO incubation, not only is the lipid diffusivity lowered, but a length scale dependent lipid diffusivity emerges, where the diffusivity deviates at lower *d* ^2^ value. Interestingly, the anomaly parameter *α* (see Eq. 7) at smaller *d* ^2^ is <1. The *D*_*app*_ values correspond to a regime where *α* is <1 as discussed earlier in the context of Eq. 2. The PSF dependent lipid diffusivity behavior is more pronounced for cholesterol containing DOPC and POPC membranes due to lipid reorganization and clustering induced by LLO oligomerization/pore formation. In comparison, for cholesterol containing DMPC membrane, LLO did not induce significant lipid re-organization, nevertheless the overall lipid diffusivity is slower and independent of PSF for the values of *d* ^2^ sampled in the experiment (Figures 7C–F). These trends are consistent with the previous confocal FCS experiments where the overall extent of change observed in *D* after exposure to LLO is the greatest for DOPC and the least for DMPC (Figures 5A–C).

The data presented in Figures 7A–C, when visualized in terms of variation of *τ*_*D*_ vs. *d* ^2^, provides a different perspective. A plot of *τ*_*D*_ vs. *d* ^2^ (Figures 7D–F) reveals the variation with the diffusion modes as a function of spot size *d*. Upon fitting the data to Eq. 10, non-zero values of intercept, *t*_0_ (positive or negative) can be connected to various forms of hindered diffusion involving presence of fluid-like nanodomains, meshwork structures or gel-like nanodomains, as shown schematically in Figure 4B. From the lipid composition dependent data as shown in Figure 7, a dynamical crossover is observed for POPC and DOPC after addition of LLO. However in the case of DMPC although *τ*_*D*_ increases for a given *d*, a dynamical crossover is not observed within the resolution of the microscope used. The length scale, *ξ* (vertical dotted line in Figures 7D–F), is slightly higher for DOPC when compared to POPC. In addition the intercept, *t*_0_, is considerably larger for DOPC when compared with POPC. AFM observations reveal that LLO pore assemblies on POPC membranes are smaller and more compact when compared to greater disorder in pores observed on DOPC membranes. These observations suggest that the spatial extent of the induced dynamic heterogeneity during pore formation in DOPC is accentuated due to the increased underlying fluidity in the membrane. Spot size variation STED-FCS experiments with DOPC:Chol SLBs with varying cholesterol content Sarangi et al. (2016a) illustrate that at lower cholesterol content (25%), dynamic heterogeneities absent in pristine bilayers emerge upon LLO addition driven by enhanced cholesterol sequestration required for LLO pore formation (see also Figure 9 and related discussion). At higher cholesterol content (33.33 and 50%) the perturbation induced due to lateral cholesterol movement during LLO pore formation is subdued, and the length scales at which a dynamic crossover occurs as a function of spot size *d* are similar to those observed in pristine bilayers. Selective leaflet tagging coupled with cholesterol incorporated in either the extracellular and cytosolic leaflets succinctly illustrate the influence of LLO binding and cholesterol sequestration that occurs in the extracellular leaflet during pore formation.

The cholesterol sequestration and transient trapping of lipids to the membrane bound motifs present in the toxins, en route to LLO oligomerization and subsequent pore formation was observed when the content of cholesterol was sub optimal (Sarangi et al., 2016a). However, at high cholesterol content in DOPC:Chol (1:1) membranes the observed reduction of diffusivity was mostly dominated by direct lipid-LLO binding instead of cholesterol enrichment or sequestration. In another report by Sarangi et al. (2017) utilizing spatially resolved STED-FCS measurements with pristine DOPC:Chol (1:1) membrane, nanoscale dynamic heterogeneities at both slow (cholesterol rich) and fast (cholesterol poor) regions were observed. Upon LLO addition to these bilayers drastic changes were observed; the dynamic crossover vanishes in regions with slow dynamics, and the crossover *ξ* as well as the proteolipidic nanodomain sizes increases in the cholesterol poor domains with fast dynamics when compared to similar domains in pristine bilayers (Sarangi and Basu, 2019). Given the generality of the FCS diffusion law plots and their utility in providing insights about the microscopic nature of membrane dynamics all subsequent plots of spot variation STED-FCS in this review will be discussed based on the *τ*_*D*_ vs. *d* ^2^ plot although the representation in terms of *D* and/or *D*_*app*_ can also be equivalently used.

**FIGURE 8:**
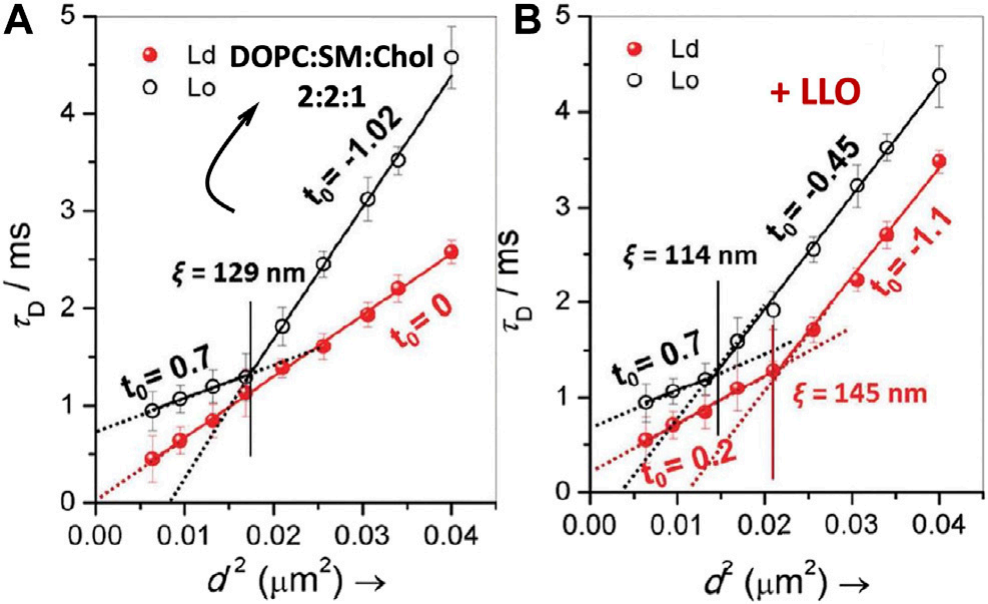
Illustrates FCS diffusion law, *τ*_*d*_ versus *d* ^2^ plots for DOPC:SM:Chol (2:2:1) in the absence **(A)** and in the presence of LLO **(B)**. In each panel, the transit time obtained from L_*o*_ and L_*d*_ regimes are represented by open and closed symbols respectively. The solid lines in the respective panels are the linear fits using Eq. 10 and the dotted lines represent the extrapolated fits to illustrate the intercepts. The vertical lines, in respective panels, demarcates the crossover length scale, *ξ*, separating different dynamical regimes. adapted from Sarangi and Basu (2018).

Sarangi and Basu (2018) used spot variation STED-FCS to study LLO binding and pore formation in ternary-domain forming membrane compositions, DOPC:SM:Chol (2:2:1). The corresponding STED-FCS data acquired from the liquid ordered, L_*o*_ and liquid disordered L_*d*_ phases before and after LLO incubation are shown in Figure 8. Apart from preferential binding to different phases, the STED-FCS data distinguished pathways for creation and annihilation of underlying nanoscale membrane nanodomains upon LLO binding and assembly. For example, in the liquid ordered, L_*o*_ phase, before LLO addition, a dynamic crossover occurred at *ξ* = 129 nm, and above and below *ξ*, the dynamics did not correspond to free Brownian diffusion (Figure 8A). These trends reflect the intrinsic gel-like nature of the *L*_*o*_ phase. However, for the L_*d*_ phase, diffusion is Brownian at all length scales observed (Figure 8A). The domain size, *ω* estimated using Eq. 11 for *t*_0_ < 0 was 87 nm.

Upon exposure to LLO, a distinct dynamic crossover was observed in the L_*d*_ phase (Figure 8B), indicating preferential binding of LLO to the L_*d*_ phase with a non-zero *t*_0_ value emerging both below and above the crossover point at *ξ* = 145 nm. Binding to the L_*d*_ phase is consistent with the experiments carried out on GUVs (Ponmalar et al., 2019), and was further supported by AFM data. In contrast the perturbation to the L_*o*_ phase upon LLO exposure was less, and the crossover point occurred at *ξ* = 114 nm. The estimated domain sizes, *ω* (Eq. 11) after LLO addition, for the L_*o*_ and L_*d*_ phases were 61 and 100 nm respectively. These results suggest the annihilation or reduction of the domain size in the L_*o*_ phase and the creation of proteolipidic nanodomains in the L_*d*_ phase induced by LLO. The preferential partitioning of LLO to the L_*d*_ phase is due to the increased availability of cholesterol even though it is present at sub-optimal levels when compared with the L_*o*_ phase. Hence the local redistribution and sequestration of cholesterol leads to the observed dynamical heterogeneity in the L_*d*_ phase. The annihilation of domain sizes in the L_*o*_ phase can be attributed to cholesterol sequestration from the L_*o*_ phase to the L_*d*_ phase. Interestingly the proteolipidic nanodomains created in the L_*d*_ phase seem to have raft-like stability supported by an invariant dynamic crossover upon cholesterol extraction by methyl-beta-cyclodextrin (M*β*CD). Cholesterol is typically depleted adding M*β*CD to the cell membranes (Mahammad and Parmryd, 2015). These observations support the hypothesis that plasma membranes of higher organisms, contain phase-separated nanodomains consisting of lipids and proteins that exist in L_*o*_/raft-like behaviour (Lingwood and Simons, 2010).

#### 4.1.3 Molecular Interactions of Lipids and Cholesterol Revealed by MD Simulations

The confocal FCS and STED-FCS experiments reveal the modulation of lipid dynamics in the vicinity of the pore complex at length scales typically above ∼50 nm. Atomistic MD simulations (Ponmalar et al., 2019; Cheerla and Ayappa, 2020) provide detailed molecular information at length scales below 15 nm to further enhance our understanding of the underlying lipid and cholesterol reorganization as a function of the state of the membrane bound, prepore or membrane inserted states. All atom MD simulations on DOPC membranes with 30% cholesterol membranes with a single D4 sub-unit placed at the membrane interface in a prepore configuration as well as simulations with a full monomer illustrate that binding of cholesterol to the undecapeptide loops result in reduced lipid diffusivity predominantly in the extracellular leaflet of the bilayer (Ponmalar et al., 2019).

**FIGURE 9:**
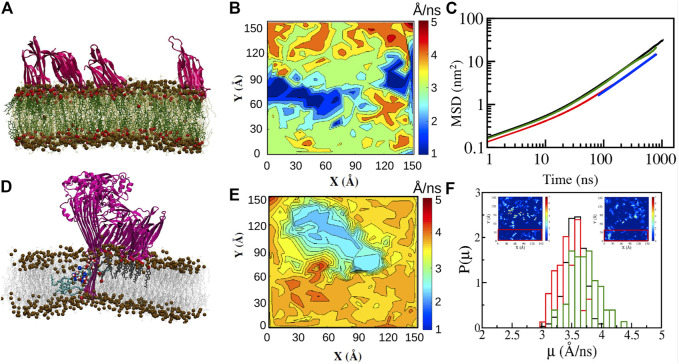
All atom simulations of LLO on DOPC:Chol (70:30) bilayers. **(A)** Snapshot (1 µs) of four D4 membrane bound sub units (pink). Cholesterol (dark green with red OH groups), DOPC (light green). Distinct cholesterol sequestration is visible below the D4 sub units **(B)** Mobility maps of the center of mass of DOPC molecules with a Δ*t* = 1 ns (Eq. 4) illustrate the lowered lipid displacements in the extracellular leaflet due to D4 binding and cholesterol **(A)** sequestration. **(C)** MSD data reveals the lowered diffusion of lipid molecules in the extracellular leaflet. **(D)** Snapshot (1 µs) of the membrane inserted tetrameric LLO arc illustrates the lipids (cyan) in a toroidal conformation, cholesterol (dark grey) bound to the D4 sub units and the water channel (oxygen—red, hydrogen—white). **(E)** Same as **(B)** for the cytosolic leaflet. **(F)** Distribution of mobilities illustrates the higher displacement for lipid molecules in regions away from the pore complex (red rectangle in the insets). The insets depict areal density of cholesterol molecules for the extracellular **(left)** and cytosolic **(right)** leaflets indicating cholesterol hot spots due to binding with the D4 sub unit. The units for the inset color bars are in 10^–2^ molecules/Å^−2^. Reprinted by permission from Springer Nature Customer Service Centre GmbH Springer Nature, Journal of Membrane Biology, Molecular Dynamics Study of Lipid and Cholesterol Reorganization Due to Membrane Binding and Pore Formation by Listeriolysin O, Cheerla and Ayappa (2020).

Lipid order parameters, lipid mobilities, and diffusion coefficients of lipid and cholesterol molecules have been analyzed in MD simulations of tetrameric oligomeric units of LLO in both the membrane bound and membrane inserted (arc-like pores) states in DOPC:Chol membranes (Cheerla and Ayappa, 2020). Similar to the trends observed with the single membrane bound units, these simulations reveal that the dominant decrease in lipid diffusivity occurs in the extracellular leaflet (Figures 9B,C,E). LLO induced spatial heterogeneity is primarily driven by local density enhancement of cholesterol in the vicinity of the protein as observed in the simulation snapshots illustrated in Figure 9A,D. This spatial heterogeneity additionally leads to distinct differences in lipid and cholesterol mobility across the two leaflets as well as enhanced lipid mobilities in regions where cholesterol is depleted (Figure 9F). This reinforces, albeit at smaller length scales, the increased lipid diffusivity due to cholesterol depletion observed in regions away from the LLO pore complex (Figure 5) in the STED-FCS experiments (Sarangi et al., 2016a) as well as the local enhancement of cholesterol concentration due to membrane binding. The periodic boundary conditions used in the MD simulations result in a high protein to lipid ratio, representative of the situation in experiments carried out in the crowded regime to induce local heterogeneities (Sarangi et al., 2016a) as well as slowed down diffusion observed at high LLO concentrations (Ilanila et al., 2021) in the SLB experiments (Figure 6B).

### 4.2 Biomembrane Dynamics in Presence of ClyA

**FIGURE 10:**
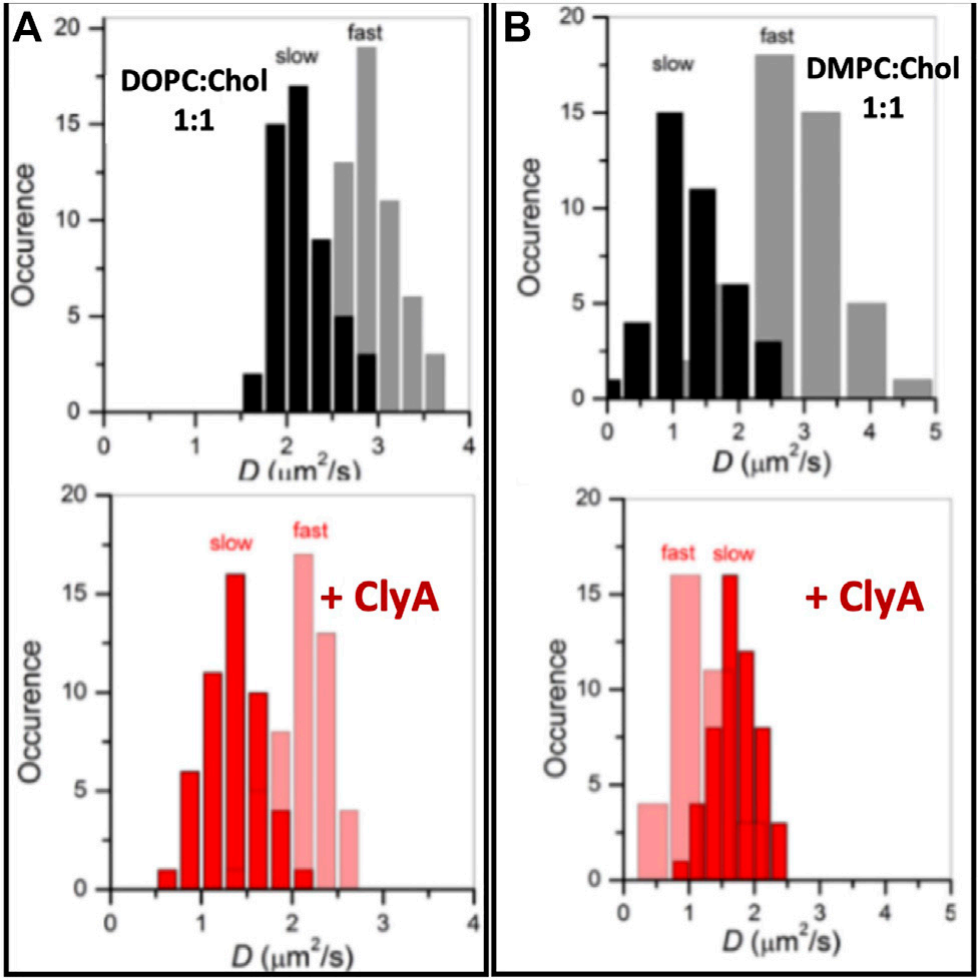
Lipid dynamics on supported lipid bilayers of two compositions DOPC:Chol (1:1) **(A)** and DMPC:Chol (1:1) **(B)** in the absence **(top)** and presence **(bottom)** labelled with Atto488DMPE. Upon ClyA interaction, the domains with fast and slow dynamics reorganised to result in slowed down lipid diffusion. Adapted from Sarangi and Basu (2019).

In this section we turn our attention to ClyA which belongs to the class of *α* toxins with a distinctly different pore formation mechanism (Sathyanarayana et al., 2020) when compared with the cholesterol dependent *β* cytolysin, LLO. We present our findings using confocal and STED-FCS measurements which probe membrane reorganization, lipid dynamics and domain preference during pore formation by ClyA. Confocal FCS measurements on SLBs of DOPC and DMPC bilayers (Sarangi and Basu, 2019) reveal distinct differences at high cholesterol concentrations of 50%. Domain formation at these high cholesterol concentrations gives rise to two distinct populations of lipid diffusivities denoted as “fast” and “slow” in pristine bilayers (Figures 10A,B) corresponding to cholesterol rich and cholesterol poor regions respectively (Sarangi et al., 2017). Diffusivity data in the DMPC:Chol (1:1) bilayer revealed slowed down lipid diffusivity in both cholesterol rich and cholesterol poor domains upon exposure to ClyA, indicating that ClyA binding and pore formation occurs in both these dynamically distinct domains. In contrast, for the DMPC:Chol (1:1) membrane, the binding of ClyA induced a decrease in the diffusivity in the cholesterol poor (fast) regions and while the opposite is observed for the cholesterol rich (slow) regions (see Figure 10B). Alongside domain mixing, confocal microscopy images unveils the formation of micron scale ClyA oligomers upon binding to the DMPC:Chol membrane, further suggesting a selective preference of ClyA to DMPC:Chol membranes compared to DOPC:Chol membranes.

Studies with a three component domain forming DOPC:SM:Chol (2:2:1) membrane by Sarangi and Basu (2018) provide additional insights into ClyA pore formation. The preferential partitioning of ClyA to the phase separated domain forming membranes, was found to be distinctly different from that of LLO. ClyA preferentially binds to the SM:Chol rich, L_*o*_ phase when compared to the L_*d*_ phase preferred by LLO. By utilizing confocal based microscopy imaging of DOPC:SM:Chol (2:2:1) monolayer and SLBs, and by AFM imaging, ClyA is seen to increased the dynamics of lipids only in the L_*o*_ phase leaving the L_*d*_ phase dynamics relatively unperturbed. This is in contrast to LLO which has the opposite effect (Sarangi and Basu, 2018).

#### 4.2.1 Nanoscale Dynamics

**FIGURE 11:**
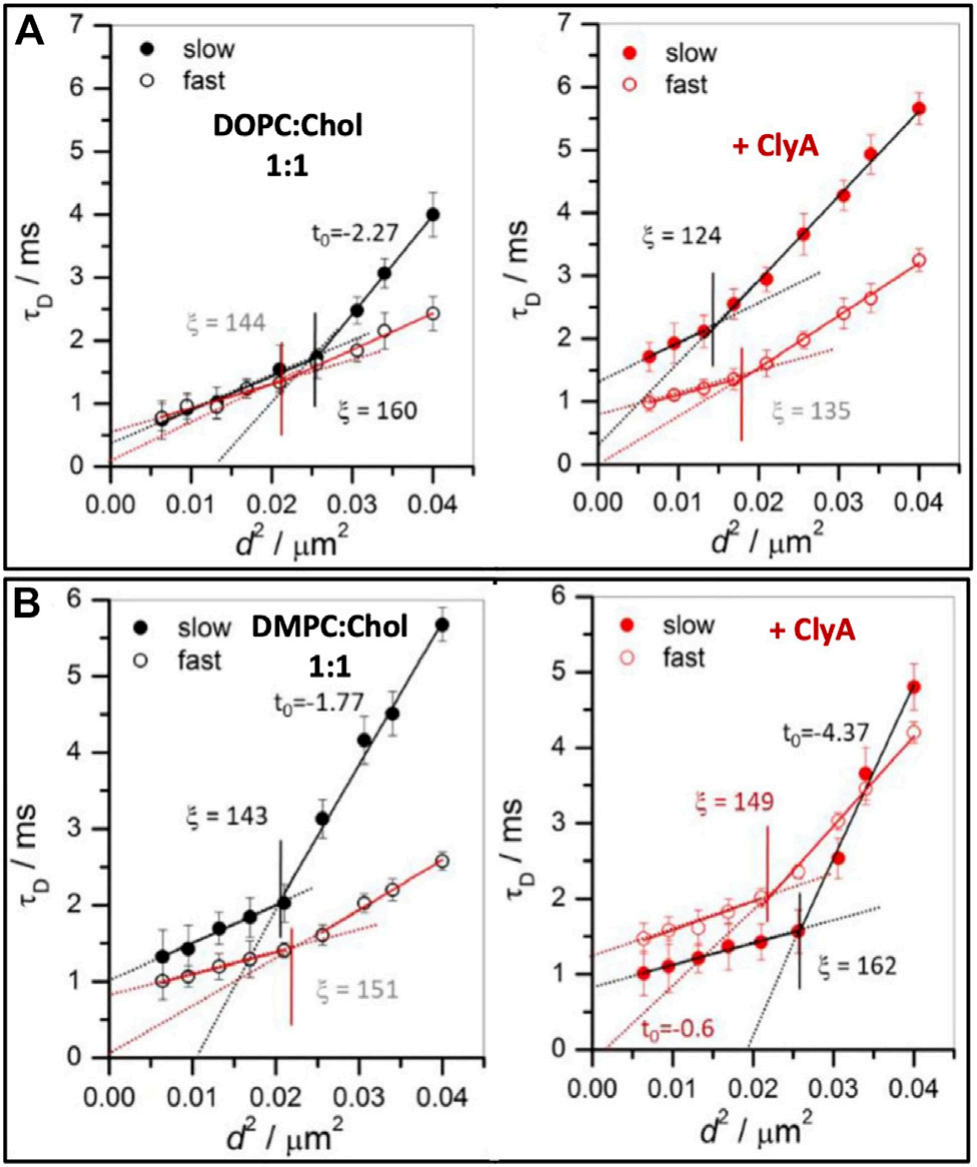
Illustrates FCS diffusion law plots, transit time (*τ*_*d*_) vs. *d* ^2^ for **(A)** DOPC:Chol (1:1) and **(B)** DMPC:Chol (1:1) SLBs before **(left panel)** and after **(right panel)** ClyA addition. The slow and fast regimes are represented by closed and open symbols. The solid lines in the respective panels are the linear fit using Eq. 10 and the dotted lines are the extrapolated line to show the intercept. The vertical lines, in respective panels, demarcates the crossover length scale, *ξ*, separating different dynamical regimes whether Brownian or non-Brownian diffusion. Adapted from Sarangi and Basu (2018).

Using STED-FCS Sarangi et al. (2017) have shown the dynamic heterogenities at both cholesterol rich (slow) and cholesterol poor (fast) regimes of DOPC:Chol (1:1) membranes. In the cholesterol poor domains, *τ*_*D*_ versus *d* ^2^ data have shown a distinct crossover at *ξ* = 160 nm above which the intercept was negative (*t*_0_ = −2.27). The estimated domain size, *ω* in this slow regime was 119 nm. Conversely, in the fast regimes, free Brownian dynamics is observed (*t*_0_ = 0) with a weak crossover occurring at *ξ* = 144 nm (see Figure 11A, left panel). Upon ClyA addition, *ξ* decreases from 160 to 124 nm in the slow regimes and the lipid dynamics above this length scale dynamics become free Brownian further supporting the notion of overall homogenizing effect occurring at the nanoscale due to ClyA pore formation. However, in regions with faster dynamics, the crossover length scale did not change significantly when compared to that of the pristine membrane (Figure 11A, left panel). This data clearly reveals the preference for ClyA to bind to the cholesterol rich regions (slower moving domains) in these cholesterol rich bilayers.

STED-FCS diffusion analyses of DMPC:Chol (1:1) membranes also reveals the presence of a dynamic crossover at the nanoscale for both the fast and slow regions (Figure 11B). In the fast (cholesterol poor) regions, free Brownian diffusion is observed above *ξ* = 151 nm while in the slow (cholesterol rich) regions deviation from free Brownian diffusion (*t*_0_ = −1.77) is observed above *ξ* = 143 nm. The estimated domain size, *ω* in the slow regime was reported to be 94 nm. Interaction of ClyA had a profound effect on both these regimes as reflected in a large increase in the intercept value (*t*_0_ = −4.37) in the slow regime and a shift from free Brownian diffusion in the fast region (*t*_0_ = −0.6). These spot variation STED-FCS measurements on cholesterol rich membranes reveal that the association and binding of ClyA is more pronounced in DMPC:Chol membranes when compared with that of DOPC:Chol membrane. These conclusions can be drawn from the greater extent of dynamic perturbation induced in the DMPC:Chol bilayers as illustrated in Figure 11B.

Cholesterol is known to have a condensing effect on unsaturated low melting lipids and a fluidizing effect on saturated high melting lipids (Krause and Regen, 2014). This therefore increases and decreases respectively, the underlying lipid fluidity in DMPC and DOPC membranes. In addition recent MD simulations (Varadarajan et al., 2020) of the dodecameric ClyA pore complex in a pure DMPC bilayer indicates the localized extent of a small hydrophobic mismatch induced by ClyA. The hydrophobic mismatch is expected to be greater for the longer tailed DOPC bilayers. The hydrophobic mismatch coupled with the condensing effect of cholesterol, correlates with the reduced pore forming propensity for ClyA in DOPC bilayers.

Spot variation STED-FCS measurements on DOPC:SM:Chol SLBs (Sarangi and Basu, 2018) bilayers reveal interesting modulations to the lipid dynamics that are not observed in the confocal-FCS measurements. For example, upon ClyA binding to the SM rich L_*o*_ phase, not only has the dynamic cross-over (observed before ClyA addition) disappeared in the corresponding *τ*_*D*_ versus *d* ^2^ data, but the intercept *t*_0_ is less negative indicative of the formation of small (40 nm) proteolipidic nanodomains in an increasingly homogeneous L_*o*_ phase. However, in the L_*d*_ phase, where the free Brownian diffusion occurs in pristine bilayers, a dynamic crossover occurs at *ξ* = 115 nm, above which the intercept is zero indicating hindered lipid diffusion due to the presence of lipidic nanoscale domains.

Therefore ClyA binding tends to homogenize the L_*o*_ phase and induce a weak dynamic crossover in the L_*d*_ phase. These trends were attributed to SM-Chol rich lipidic nanodomain migration from L_*o*_ to L_*d*_ phase manifested by ClyA binding. Furthermore, the emergence of these nanodomains in their respective phases are susceptible to cholesterol extraction by M*β*CD, indicating that these domains do not possess raft-like stability unlike that observed in proteolipidic nanodomains formed by LLO (Sarangi and Basu, 2018) and discussed in the previous Section 4.2. Thus ClyA seems to alter the compositional lipid landscape and consequently associated dynamic heterogeneities in the different co-existing phases. The overall effect of ClyA on various membrane compositions showed that availability of cholesterol and the fluidity of lipid membrane systems are critical to obtain strong membrane binding as well as modulating membrane reorganization at the nanoscale. Additional insights into cholesterol mediated ClyA binding are obtained from single particle tracking experiments and MD simulations discussed next.

#### 4.2.2 Molecular Dynamics Simulation of ClyA

**FIGURE 12:**
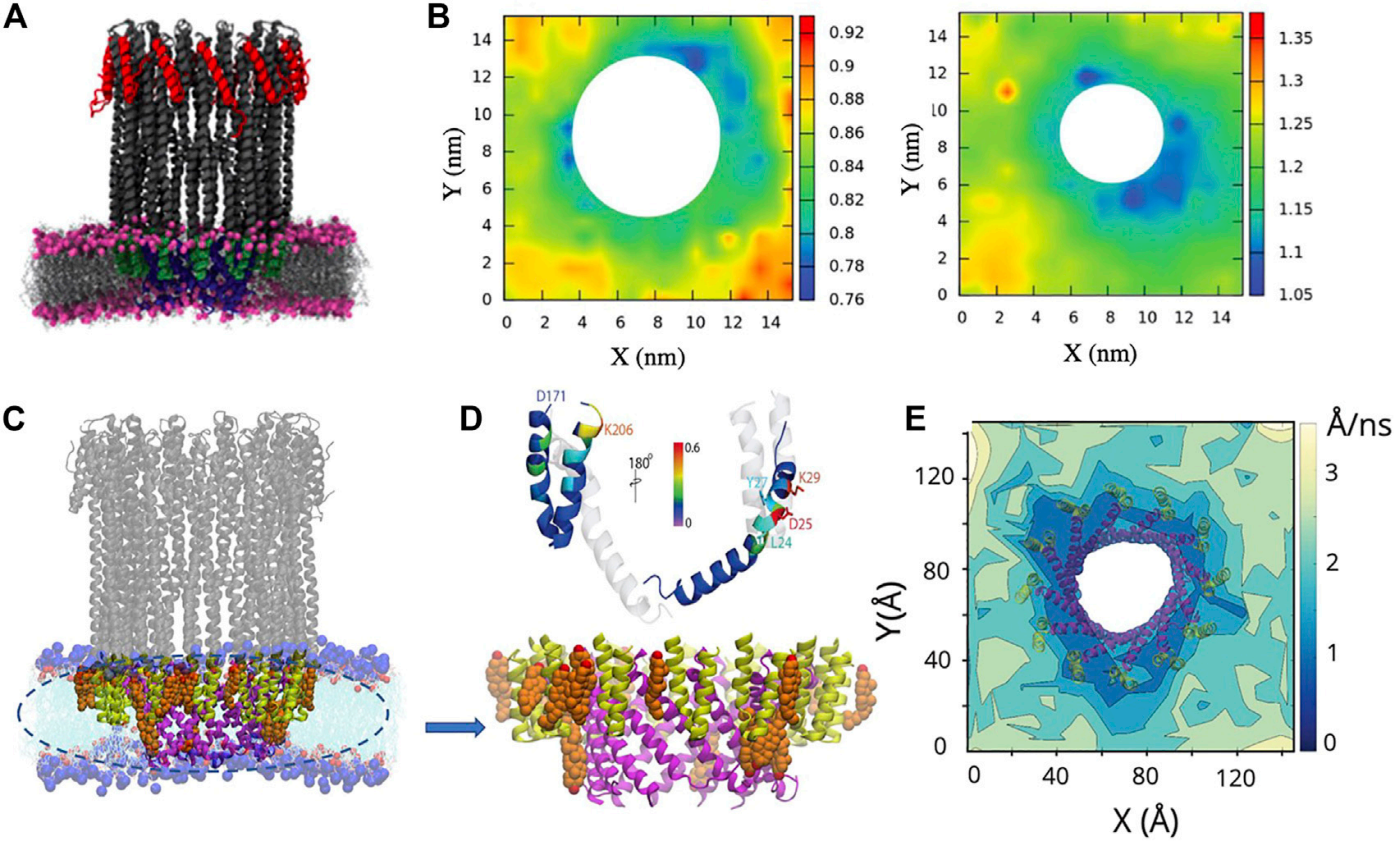
Results from all atom MD simulations of the dodecameric ClyA pore complex. **(A)** Snapshot of the ClyA pore in a pure DMPC bilayer. **(B)** Particle displacement maps (Δ*t* = 50 ns) of the extracellular **(left)** and cytosolic leaflet **(right)**. Increased lipid fluidity is observed in the cytosolic leaflet. Color bars are in units of nm. **(C)** Snapshot of the ClyA pore in a DOPC:Chol (70:30) membrane. Cholesterol (orange) with OH groups (red), membrane inserted *β* tongues (green), membrane inserted *α* helices (pink), lipids (cyan). **(D)** Cholesterol binding sites on the membrane inserted *β* tongue, helix-loop-helix motif and the *N* terminus *α* helix **(top)**. Expanded view of the membrane inserted portion of the ClyA pore illustrating cholesterol binding sites located between two membrane inserted *α* helices **(bottom)**. **(E)** Cholesterol mobility map (Δ*t* = 1 ns) illustrating the lowered dynamics of cholesterol in the vicinity of the pore complex. **(A,B)** Reproduced from Varadarajan et al., 2020; **(C-E)** Reproduced from Sathyanarayana et al., 2018.

Using fluorescently labelled ClyA on POPC supported lipid bilayers, single particle tracking measurements with total internal reflection fluorescence (TIRF) experiments show the distinct slowing down of mobile ClyA protomers on the membrane induced by the presence of cholesterol (Sathyanarayana et al., 2018). In the presence of cholesterol, the conversion to slower moving membrane bound ClyA populations was used as a signature of the complete insertion of the N-terminus into the membrane. All atom MD simulations reveal cholesterol binding sites in the N-terminal which enhances binding to the membrane prior to assembly (Figure 12D). Upon pore formation cholesterol binding occurs in the *β*-tongue pockets formed between adjacent membrane inserted protomers in the ClyA pore complex as illustrated in Figures 12C,D. Both lipid and cholesterol lateral displacements (Figure 12E) are retarded around the pore complex and these simulations reveal the stabilizing influence of cholesterol on the pore formation pathway for ClyA, supported by the enhanced pore formation kinetics in both RBC lysis and vesicle leakage experiments (Agrawal et al., 2017; Sathyanarayana et al., 2018). The role of cholesterol in ClyA pore formation has also been confirmed in vesicle leakage experiments by Peng et al. (2019). Thus the propensity of ClyA to bind to cholesterol rich domains in the high cholesterol containing DOPC and DMPC bilayers could be attributed to the increased stability imparted to the membrane inserted oligomeric complex as illustrated in Figures 12C,D.

**FIGURE 13:**
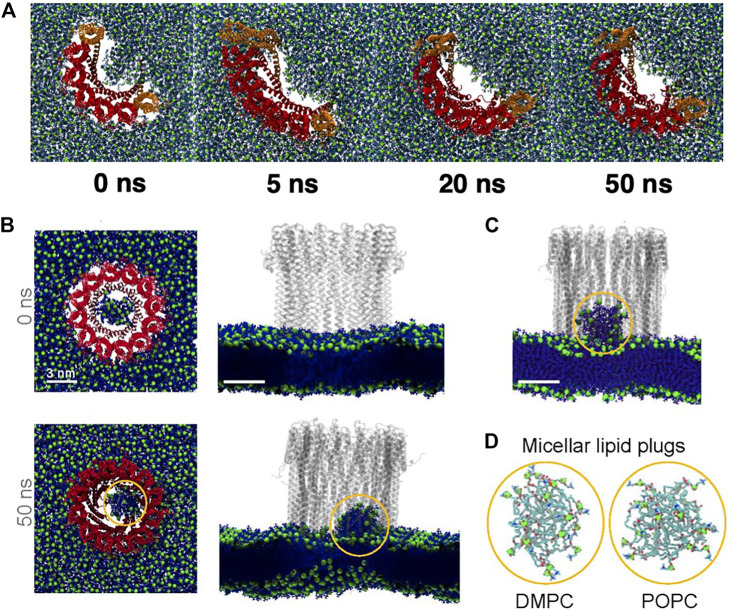
Snapshots from all atom MD simulations of ClyA in lipid bilayers illustrating arc formation **(A)** and lipid ejection events **(B**–**D)**. **(A)** Dynamics of lipid evacuation and formation of a water channel (white) for a 7-mer membrane inserted ClyA arc. Formation of a micellar lipid plug in a dodecameric ClyA pore in a DMPC membrane. **(B)** top views, and corresponding side views. **(C)** Micellar plug formation in a POPC membrane (50 ns). In all cases water molecules have not been shown for improved clarity. **(D)** Snapshots of the micellar plugs. **(A)** Reproduced with permission from Desikan et al. (2017), Copyright (2017), **(B)** Reproduced from Desikan et al. (2020).

MD simulations reveal several molecular insights into lipid and cholesterol structural and dynamic alterations during ClyA pore formation. All atom MD simulations with ClyA arcs in phospholipid bilayers without cholesterol, illustrate the reorientation of lipids to form a toroidal edge in order to stabilize transmembrane water channels (Desikan et al., 2017). Lipid reorientation and displacement to stabilize the pore channel took place within 50–60 ns with the different lipids examined (DMPC and POPC). Measurement of the lipid survival probabilities in the growing pore lumen initially occupied with lipids (Figure 13A) indicated a fast time constant associated with the displacement of lipids with a slower time constant due to reorientational relaxation of the lipid while forming the toroidal edge. Faster evacuation kinetics were observed for the POPC lipids when compared with DMPC. Simulations also reveal the ability of a single membrane inserted protomer to stabilize a water channel lending evidence for the growing pore pathway for ClyA (Desikan et al., 2017; Sathyanarayana et al., 2018). Insertion of the complete dodecameric pore into the membrane (Desikan et al., 2020; Desikan et al., 2021) using both all atom and a coarse grained Martini model, led to the formation of a micellar aggregate which rises towards the extracellular space, indicative of a lipid removal pathway in the event of a direct prepore to pore transition. The formation of a lipid aggregate for DMPC and DOPC lipid membranes using all atom MD simulations are illustrated in Figures 13B–D.

MD simulations of ClyA and *α*-hemolysin (AHL) pores in pure DMPC bilayers contrast the influence of membrane inserted *α* helices in the case of ClyA with the membrane inserted *β* barrel for AHL (Varadarajan et al., 2020). Analysis of the lipid tilt angle and chain order parameters that surround the pore complex indicate a short ranged perturbation of up to 2.7 nm on lipid structure. Lipids form a tightly bound shell around the pores and retarded lipid dynamics extend to over 4 nm from the lipid-protein interface (Figures 12A,B). The retardation in lipid dynamics is greater in the extracellular leaflet when compared with the increased disorder induced in the cytosolic leaflet of the bilayer (Figure 12B). Analysis of distributions of lipid displacements around the vicinity of pore, show long tails revealing a distinctly non-Gaussian nature (Varadarajan et al., 2020), a signature of the induced dynamic heterogeneity due to membrane insertion, confirming albeit at smaller length scales, the observed dynamic variations observed in spot variation STED-FCS experiments with ClyA (Sarangi and Basu, 2018).

### 4.3 Other Pore-Forming Toxins

We have extensively reviewed the changes observed on lipid dynamics and membrane reorganization that occurs during the pore formation the *β*-PFT, LLO and the *α*-PFT, ClyA. In this section, we highlight studies concerned with other PFTs and their influence on lipid dynamics.

#### 4.3.1 *α*-PFTs

Cholera toxin, an *α*-PFT, is secreted by the bacteria *Vibrio cholerae*. It has been used as a raft detecting molecule due to it’s preferential binding to ganglioside (GM) lipids present on the ordered domains of the plasma membrane (Kenworthy et al., 2000). The cholera toxin is known to perturb lipid packing due to lipid receptor (GM) clustering which can result in the formation of new lipid phases (Watkins et al., 2011). The changes in lipid order upon interaction of cholera toxin binding domain was investigated by Sun et al. (2015) using coarse grained dissipative particle dynamics (DPD) simulations to established the local disordering of lipids due to clustering of GM lipids. Using FCS, reduced lipid dynamics was observed in regions away from protein binding raft regions due to suppression of the formation of percolating *L*_*α*_ regions above the main DMPC phase transition temperature (Forstner et al., 2006). Confocal FRAP measurements on live COS-7 cell lines reveal the lowered diffusion of the bound cholera toxin and illustrate that the diffusion of several cell surface markers are unaltered due to cholera toxin binding (Day and Kenworthy, 2012). The lowered mobility of bound cholera toxin was influenced by ATP driven actin cytoskeleton reorganization. Based on the diffusion measurements with cell surface markers, the findings by Day and Kenworthy (2012) indicate that plasma membrane re-organisation was not significantly perturbed due to GM lipid clustering, in contrast to observations in model membrane SLB platforms (Forstner et al., 2006). Additionally, GM1 clustering has been reported to induce membrane bending which could be the early indicator of pathogenic endocytosis (Kabbani et al., 2020).

Using time lapse 3D live cell imaging, FRAP, FRET and FCS (García-Sáez et al., 2011) the lipid dynamics of model membranes as well as mammalian cells were investigated upon treatment by equinatoxin II, a sphingomyelin specific PFT secreted by the sea anemone *Actinia equina*. Extensive plasma membrane reorganization was observed based on enhanced lipid diffusion kinetics measured using FRAP. This results in the formation of microscopic domains that resemble coalesced lipid rafts which might be one of the mechanisms by which host cells evade PFT attack. When lipid bilayers of different lipid compositions were analysed, the presence of phase separated domains enhanced the pore formation mechanism of equinatoxin II (Barlič et al., 2004). Interestingly, the activity of equinatoxin is also observed to be dependent on the lipid composition as revealed in neutron reflectivity measurements (Wacklin et al., 2016). In a pure DMPC lipid bilayer, the protein interacts weakly with the membrane, whereas in the presence of SM and cholesterol, insertion was prominent with faster pore formation kinetics indicating the importance of lipid phase-separation and domain formation in the pore formation pathway of equinatoxin II.

Diphtheria toxin was investigated to identify the insertion mechanism and the role of lipids during the translocation of proteins (Chenal et al., 2009). Combining the results from specular neutron reflectometry and solid-state NMR spectroscopy, they conclude that the T-domain of the protein induces disorder in the surrounding lipids resulting in creation of a water channel at pH 4. This destabilisation of the membrane by increasing the local disorder could facilitate the insertion and translocation of the catalytic domain of the toxin into the cellular regions. Similar to diphtheria toxin, colicin N was also reported to require additional aid from an external outer membrane protein, OmpF. The time dependent neutron reflectivity data coupled with selective deuteration revealed that OmpF mediates the insertion of the protein in to lipid monolayers at the air-water interface (Clifton et al., 2012).

#### 4.3.2 *β*-PFTs

Pneumolysin (Ply) a *β*-PFT that falls under the category of CDCs, is structurally similar to LLO. Martini based coarse grained MD simulations on the inserted states of Ply revealed the formation of lipid arcs and lipid ejection by the formation of a micellar aggregate (Vögele et al., 2019) due to the insertion of fully formed pores. Using Raman spectroscopy on optically trapped large unilamellar vesicles (LUVs), the effect of Ply on the short range order and rotational diffusion of lipids was investigated (Faraj et al., 2020). Although changes in the lipid packing and lipid order were not observed during the pore formation, a phase transformation from L_*o*_-like to L_*d*_-like lipid domains with the formation of microdomains surrounding the pores were observed.

In case of lysenin, a *β*-PFT that specifically binds to the sphingomyelin (SM), high speed AFM imaging revealed the reorganisation and kinetics of pore formation on supported lipid bilayers. Yilmaz and Kobayashi (2015) observed that lysenin initially binds to the SM-rich L_*o*_ domain and with increasing protein concentration, pore formation gradually expands into the L_*d*_ phase, to cover the entire membrane. The line tension that exists between the L_*o*_ and the L_*d*_ domain was not affected by lysenin, however Ros et al. (2013) reported that sticholysin, another SM binding PFT, has a tendency to reduce the line tension with a tendency to induced lipid mixing. The influence of line tension mediating proteins on domain morphology has recently been reviewed by Bodosa et al. (2020).

## 5 Discussion

PFTs give rise to a unique class of protein membrane interactions due to the presence of a large extracellular region present in the assembled pore state as well as the distinct secondary structures present in the transmembrane regions of the pore complex. The transient and dynamic nature of the pore forming process coupled with receptor mediated membrane reorganization induces dynamic heterogeneity in the membrane, driven in part by lipid rearrangement and ejection events unique to PFT pore formation. In this review, we primarily focus on the modulation of the underlying lipid dynamics and molecular insights gleaned from both confocal and STED based fluorescence microscopy measurements and MD simulations which collectively shed light on the complex pore formation pathways of PFTs. Monitoring the lipid dynamics offers distinct insights into the pore forming mechanisms as influenced by the specific phases involved in the pore formation process in multicomponent membranes. We point out that interrogating the dynamical and compositional heterogeneity induced to the membrane complements, for example, AFM imaging studies on PFTs.

Monitoring lipid diffusion reveals information on the different bound oligomeric states of the *β*-PFT, LLO. Confocal FCS studies coupled with FRET reveal that the lowered lipid diffusivities observed on GUVs correlate with membrane bound states with an increase in diffusivities resulting from membrane inserted states. All atom MD simulations shed additional light on these variations, where the membrane bound D4 sub units of the LLO protein result in reduced lipid diffusivities and enhanced fluidity is observed for the membrane inserted states (Ponmalar et al., 2019; Cheerla and Ayappa, 2020). Thus membrane binding and insertion which are key steps in the PFT oligomerization and pore formation pathways have a distinct influence on the underlying lipid dynamics. The counterintuitive non-monotonic variation in lipid diffusivity as a function of LLO protein concentration provides several novel insights into membrane reorganization during pore formation (Ilanila et al., 2021). Coupled with a free area based diffusion model, the initial increase in diffusivity is associated with a membrane populated with ring-like structures and the lowered diffusivity at higher protein concentrations where the membrane is populated with arc-like structures and pores. An important aspect of the CDC family of pore forming toxin is the necessary role of cholesterol during pore formation.

An important feature of pore formation by CDCs as observed in our studies with LLO is the lipid and cholesterol reorganization induced during pore formation. STED-FCS and MD simulations show that cholesterol binding to the membrane associated D4 sub units of the LLO monomer leads to a depletion of cholesterol away from the pore complex giving rise to two sub-populations of lipid diffusivities. This induced dynamic heterogeneity due to compositional variations is accentuated at lower cholesterol content in DOPC:Chol membranes with anomalous diffusion observed at length scales below 100 nm indicative of protein induced sub-diffusive lipid nanodomains. Leaflet specific tagging, feasible in the Langmuir-Blodgett technique for SLB preparation, reveals that the extracellular leaflets are perturbed to a greater extent during LLO pore formation. Additionally, the ability to sequester cholesterol and hence effectively form pores is greater for low melting unsaturated lipids such as DOPC and POPC when compared with the higher melting unsaturated DMPC lipids (Sarangi et al., 2016a; Sarangi et al., 2016b).

**FIGURE 14:**
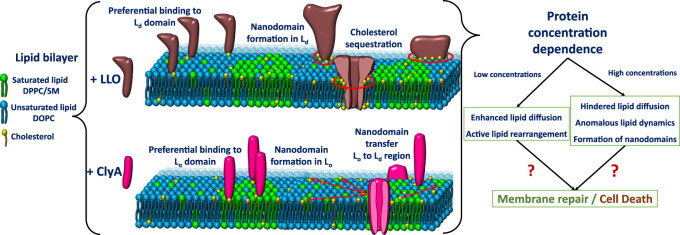
Schematic representation of the phase separated liquid ordered (L_*o*_) and liquid disordered (L_*d*_) phases on lipid bilayer membranes and the preferential binding and assembly pathways of LLO and ClyA to these domains.

Insights from STED-FCS and confocal measurements shed light on cholesterol access and differential pore forming attributes for *α*- and *β*-toxins driven by domain formation and compositional variations therein in multicomponent lipid membranes. Preferential binding of LLO to the L_*d*_ domains when compared with the cholesterol rich L_*o*_ domains indicates that despite the lower cholesterol content in the L_*d*_ domains, cholesterol is more accessible for membrane binding and insertion of the undecapeptide loops which contain the primary cholesterol recognizing motifs. Thus these events facilitate the preferential sequestration of cholesterol around the pore assemblies in the L_*d*_ phase (Figure 14). Furthermore, the increased lipid mobility in the L_*d*_ domains could facilitate membrane insertion of the *β* sheets during pore formation when compared with the lowered fluidity in the L_*o*_ domains. ClyA on the other hand preferentially binds to the SM and cholesterol rich L_*o*_ domains indicating that insertion of the *β* tongue and the subsequent conformational changes that occur during pore formation are facilitated by the higher cholesterol content in these domains (Figure 14). Although increased cholesterol has been shown to decrease ClyA binding as inferred from single particle tracking TIRF experiments on POPC:Chol bilayers (Sathyanarayana et al., 2018), the presence of cholesterol enhances pore formation in vesicle leakage experiments. MD simulations have illustrated the stabilizing role played by cholesterol in the pore assemblies of ClyA where the *β* pockets formed between adjacent protomers are favorable cholesterol binding sites.

In contrast to LLO, where the cholesterol to bound protomer ratio is large as revealed in MD simulations (Ponmalar et al., 2019; Cheerla and Ayappa, 2020) the cholesterol requirement for ClyA pore formation is significantly lower. Additionally ClyA is known to form pores in the absence of cholesterol Sathyanarayana et al. (2018). This difference is further accentuated by the larger LLO pore assemblies. Hence the requirement for not only cholesterol being present, but the underlying mobility of cholesterol plays an important role in LLO pore formation which is not the case for ClyA. M*β*-CD treated bilayers clearly reveal lowered solubility and diminished cholesterol removal due to stronger affinity of cholesterol in LLO bound membranes when compared with the increased solubility of the weakly bound cholesterol in membranes with ClyA (Sarangi and Basu, 2018). This detailed domain specific insights and differences between *β*- and *α*-PFT pore formation emerge from the resolution of lipid dynamics from STED-FCS experiments at length scales below 200 nm, combined with all atom MD simulations of the membrane bound oligomeric complex.

One of the open questions in the PFT pathway is related to the resulting fate of lipids during pore formation. Recent confocal FCS measurements on SLBs in our laboratory provide direct evidence for the loss of lipids upon LLO pore formation (Ilanila et al., 2021) as revealed in the decreasing labelled lipid fluorescence intensity upon an increase in protein concentration. It is now well accepted that LLO as well as ClyA can exist as transmembrane arc-like pores or fully inserted rings that follow a prepore insertion pathway (Ruan et al., 2016; Roderer and Glockshuber, 2017). The insertion of rings results in a loss of lipids due to ejection via the formation of a lipid micellar structure as shown in recent MD simulations with Ply another CDC of the same family as LLO (Vögele et al., 2019). This mechanism of lipid loss due to micellar aggregate formation, upon membrane insertion of the pore complex is more universal and has recently been shown to occur in smaller pores (see Figures 13B-D) formed by the *α*-toxin ClyA (Desikan et al., 2020). In contrast the formation of arcs leads to the displacement of lipids during the formation of toroidal lipids to stabilize the water channel (Desikan et al., 2017; Vögele et al., 2019; Cheerla and Ayappa, 2020).

Interestingly, the lipid response is unique not only towards different classes of PFTs, but also to the different structural and oligomeric units of the proteins as depicted in Figure 6. This indicates that the host cell response for membrane repair and toxin expulsion might depend on the conformational and oligomeric states of the toxins. Such repair mechanisms could also be triggered due to active rearrangement of lipids (marked by increased lipid diffusion) at low toxin concentrations present on the host cell apart from the traditional signalling pathways (Ilanila et al., 2021). These active rearrangement may also act as a double edged sword by facilitating kinetics of pore formation on one hand or allow for larger membrane related topological changes to enable expulsion of the toxin bound regions. Our combined confocal and STED-FCS measurements reveal significant rearrangement on model membrane platforms as a result of PFT interaction (Sarangi et al., 2016a; Sarangi and Basu, 2018; Ponmalar et al., 2019; Cheerla and Ayappa, 2020). When compared with the live cell membranes that are rich in the integral membrane proteins along with other membrane associated molecules, model membrane are relatively static and ignore the effects of dynamic cytoskeletal rearrangements and other downstream signalling mechanisms associated with cellular repair pathways (Husmann et al., 2009; Keyel et al., 2011; Atanassoff et al., 2014; Etxaniz et al., 2018). Interpreting the diffusion kinetics involving live cells using superresolution techniques like STED-FCS and single particle tracking, although more challenging, is essential for a complete understanding of the lytic pathways of PFTs.

## 6 Conclusion

We have extensively reviewed two archetypical *α*- and *β*-PFTs, ClyA and LLO respectively and addressed the mechanism of pore formation with particular emphasis towards the dynamic modulations imposed on the membrane during pore formation. In addition, we briefly discuss the relevant literature on other PFTs were lipid and membrane reorganization has been the focus. We evaluate the information obtained from fluorescence microscopy techniques at both confocal and STED resolutions which collectively span length scales ranging from 50–200 nm. We conclude that each PFT is unique in their action and highlight basic differences that arise from the membrane inserted secondary structure and receptors implicated during pore formation. Lipid dynamics and compositional variations are specific towards oligomeric states as well as conformational changes that are ubiquitous during pore formation. Typical response that occurs in lipid bilayers during PFT are reflected in cholesterol clustering induced nanodomain formation, domain coalescence, binding to specific domains and dynamical heterogeneities induced as a consequence at the nanoscale. STED-FCS experiments allow one to probe this dynamical heterogeneity and deviations from Brownian diffusion that occur due to pore formation in homogeneous two component or domain forming three component systems. With advanced computing platforms, MD simulations has emerged as a powerful tool to complement our understanding of the molecular reorganizational events that take place upon membrane binding and pore formation. Hence phenomenon such as a cholesterol binding during ClyA pore formation, lipid ejection mechanisms and the extent of induced variation in lipid dynamics during pore formation are revealed at the molecular scale. Thus a combination of experiments and simulations provides several insights into the uniquely evolved membrane disruptive pathways of PFTs.

The techniques and methods described in this review are general and are expected to be relevant to the study of other membrane interacting molecules such as peripheral and integral membrane proteins, peptides, antibiotics, synthetic dendrimers and polymers. Similar techniques can also be extended to live cell imaging to account for cytoskeletal modulations and active membrane repair mechanisms. Our review provides detailed descriptions on both techniques as well as observations that could potentially be useful in future research to explore the use of suitably modified PFTs or other protein molecules as potential protein based therapeutics platforms to mitigate and disrupt PFT based virulence pathways.
